# Combinatorial effects of environmental parameters on transcriptional regulation in Saccharomyces cerevisiae: A quantitative analysis of a compendium of chemostat-based transcriptome data

**DOI:** 10.1186/1471-2164-10-53

**Published:** 2009-01-27

**Authors:** Theo A Knijnenburg, Jean-Marc G Daran, Marcel A van den Broek, Pascale AS Daran-Lapujade, Johannes H de Winde, Jack T Pronk, Marcel JT Reinders, Lodewyk FA Wessels

**Affiliations:** 1 Information and Communication Theory Group, Department of Mediamatics, Delft University of Technology, Mekelweg 4, 2628 CD, Delft, the Netherlands; 2 Industrial Microbiology section, Department of Biotechnology, Delft University of Technology, Julianalaan 67, 2628 BC Delft, the Netherlands; 3 Bioinformatics and Statistics, Department of Molecular Biology, The Netherlands Cancer Institute, Plesmanlaan 121, 1066 CX, Amsterdam, the Netherlands; 4 Kluyver Centre for Genomics of Industrial Fermentation, the Netherlands

## Abstract

**Background:**

Microorganisms adapt their transcriptome by integrating multiple chemical and physical signals from their environment. Shake-flask cultivation does not allow precise manipulation of individual culture parameters and therefore precludes a quantitative analysis of the (combinatorial) influence of these parameters on transcriptional regulation. Steady-state chemostat cultures, which do enable accurate control, measurement and manipulation of individual cultivation parameters (e.g. specific growth rate, temperature, identity of the growth-limiting nutrient) appear to provide a promising experimental platform for such a combinatorial analysis.

**Results:**

A microarray compendium of 170 steady-state chemostat cultures of the yeast *Saccharomyces cerevisiae *is presented and analyzed. The 170 microarrays encompass 55 unique conditions, which can be characterized by the combined settings of 10 different cultivation parameters. By applying a regression model to assess the impact of (combinations of) cultivation parameters on the transcriptome, most *S. cerevisiae *genes were shown to be influenced by multiple cultivation parameters, and in many cases by combinatorial effects of cultivation parameters. The inclusion of these combinatorial effects in the regression model led to higher explained variance of the gene expression patterns and resulted in higher function enrichment in subsequent analysis. We further demonstrate the usefulness of the compendium and regression analysis for interpretation of shake-flask-based transcriptome studies and for guiding functional analysis of (uncharacterized) genes and pathways.

**Conclusion:**

Modeling the combinatorial effects of environmental parameters on the transcriptome is crucial for understanding transcriptional regulation. Chemostat cultivation offers a powerful tool for such an approach.

## Background

The transcriptional program of a cell is to a large extent determined by its extracellular environment. Signaling pathways, transcription factors (TFs) and chromatin remodeling mediate the transcriptional response that enables the organism to adapt to changed conditions. In order to understand the transcriptional response to changes in the extracellular environment, a large majority of the transcriptome analysis studies are based on the comparison of a single "reference" condition against a different condition. Genes that show a different transcript level between the two situations are often labeled "upregulated" or "downregulated" in the non-reference situation. This binary mode of analysis does not take into account the fact that many genes are influenced by multiple environmental stimuli and regulated by multiple TFs. The rate of transcription of a gene is, in general, the net result of the integration of multiple inputs. Consequently, transcriptional responses to individual environmental stimuli may be strongly dependent on the experimental context in which they are studied.

While the context dependency of transcriptional responses has been acknowledged as an important factor by several authors (e.g. [1,2]), it is only rarely considered in experimental design and in data interpretation. Three main reasons can be identified for this omission. First, most transcriptome studies on micro-organisms are based on shake-flask cultivation, in which key physiological parameters such as the specific growth rate and nutrient availability change continuously and cannot be adequately controlled. This makes it impossible to quantify the context dependency of transcriptional responses. Secondly, research questions are often approached from a one-dimensional perspective, in which differential gene expression is completely attributed to the difference between a condition of interest and a reference condition. This strategy is implicitly incorporated into the two-channel microarray experimental design, where the ratio of intensities from the channels represents the gene expression ratio between the condition of interest and the reference condition. A final factor that complicates meaningful combinatorial analyses of transcriptional regulation is that integration of data from different studies and laboratories may be hampered by differences in experimental procedures for microarray experiments (including the use of different microarray platforms, mRNA extraction, normalization and summarization algorithms [3,4]).

The "one-dimensional" design of transcriptome studies, as outlined above, ignores combinatorial effects of growth parameters, i.e., the possibility that repetition of the measurements in, for example, a different medium composition or temperature, might yield a different transcriptional response to the same change in the parameter of interest. Recently, a relatively small number of studies have quantitatively explored the context dependency of transcriptional regulation in chemostat cultures of the yeast *Saccharomyces cerevisiae *[5-8]. In steady-state chemostat cultures, individual environmental parameters can be manipulated in a controlled manner and at a fixed specific growth rate [9,10]. This forms an important advantage over the use of shake flasks and other batch cultivation procedures, in which changes in environmental parameters affect specific growth rate, thus precluding the dissection of primary responses to environmental parameters and indirect effects of a different specific growth rate. Recent chemostat-based studies have demonstrated that, indeed, specific growth rate itself has a strong effect on transcriptional regulation in *S. cerevisiae *[8,11,12]. Additionally, chemostat experiments on combinatorial effects of macronutrient limitation, oxygen availability and temperature provided compelling evidence for the impact of context dependency [5,6,13].

The goal of the present study is to quantify the influence of cultivation parameters on gene expression and specifically focus on the influence of combinatorial (or context-specific) effects of the cultivation parameters. To this end, we have compiled a microarray compendium of well-defined chemostat cultivations of yeast and employed a computational framework to analyze the effect of the cultivation parameters on gene expression. The compendium of chemostat-based transcriptome datasets is comprised of 170 microarray measurements, which have been performed over the past years in the Kluyver Centre's yeast research programme. These measurements, the majority (111 out of 170) of which have been previously published separately, encompass 55 unique growth conditions with (mostly three) independent biological replicates for each condition. Across the 55 different conditions, there are ten varying cultivation parameters, such as growth-limiting substrate, specific growth rate, aeration, pH and temperature. A forward step-wise regression model was designed and applied to quantify the (combinatorial) effect of individual environmental parameters on transcriptional regulation. This strategy is based on the assumption that the observed difference in the transcript level of a gene between two microarrays can be fully attributed to the difference in environmental parameters (and measurement noise) between these arrays. The results show that mainly due to the accurate control and measurement of the growth parameters enabled by steady-state chemostat cultivation, this assumption holds to a large degree. By employing these results from the regression analysis, we explore the significance of context dependency throughout the compendium. Its applicability for functional analysis of (uncharacterized) genes and pathways is demonstrated using the inferred causal relationship between environmental parameters and gene expression.

## Results and discussion

This section starts by describing the steady-state chemostat microarray compendium and the regression analysis to assess the influence of cultivation parameters on gene expression. Then, the combinatorial effects of cultivation parameters on the transcriptome are investigated using enrichment tests and through biological interpretation of these effects on genes of functional categories and biochemical pathways. To demonstrate the usefulness of the compendium, this section concludes by presenting two case studies concerned with, firstly, the functional analysis of uncharacterized and dubious genes, and secondly, the interpretation of shake-flask-based transcriptome studies using the compendium.

### Inferring the influence of cultivation parameters on gene expression

**Figure 1:**
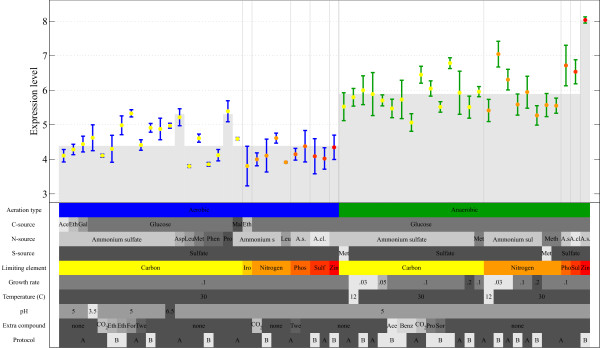
**Expression levels of *UPC2* across the 55 cultivation conditions**. The colored matrix is a schematic representation of the settings of the ten cultivation parameters over the 55 conditions. The colored lanes indicate the cultivation parameters that are employed to order the experiments, in this case, aeration type and limiting element. The applied regression model was able to explain 71% of the variance in the expression of this gene. The model selected one significant single effect, i.e. aeration type, and two significant combinatorial effects, i.e. aeration type anaerobic together with limiting element zinc and the usage of proline or asparagine as nitrogen source. The reconstructed expression pattern based on these three effects is indicated by the shaded area.

The *Saccharomyces cerevisiae *laboratory reference strain CEN.PK 113-7D (*MAT*a) was grown at steady state in chemostat cultures under 55 different conditions. A condition can be characterized by a specific configuration of the settings of ten different cultivation parameters. One of these cultivation parameters is the available carbon source. Throughout the compendium five different carbon sources were used, i.e. acetate, ethanol, galactose, glucose and maltose. Thus, these five compounds form the settings that the cultivation parameter carbon source can assume. Table 1 provides an overview of the settings for all cultivation parameters. Figure 1 depicts the expression levels of the gene *UPC2 *across all 55 conditions. The lower part of this figure is a schematic representation of the settings of the ten cultivation parameters over all conditions. Note that the expression levels are absolute expression levels that come from a single-channel microarray system and not relative expression levels, where a reference condition is employed. A regression model was designed to assess the influence of the cultivation parameters on gene expression. The model was applied to all differentially expressed genes individually. (A large majority (6005 of 6383) of the genes in the *S. cerevisiae *genome was found to be differentially expressed in at least one of the 55 conditions.) Using a step-wise approach, the regression model iteratively selects significant predictors in order to reconstruct the expression pattern of a gene.

**Table 1:** Settings within the cultivation parameters

Aeration type	C-source	N-source	S-source	Limiting element
Aerobic	Acetate (Ace)	Ammonium chloride (A.cl.)	Methionine (Met)	Carbon
Anaerobic	Ethanol (Eth)	Ammonium sulfate (A.s.)	Sulfate	Iron (Iro)
	Galactose (Gal)	Asparagine (Asp)		Nitrogen
	Glucose	Leucine (Leu)		Phosphorus (Pho/Phos)
	Maltose (Mal)	Methionine (Met/Meth)		Sulfur (Sul/Sulf)
		Phenylalanine (Phen)		Zinc (Zin)
		Proline (Pro)		

Growth rate	Temperature (C)	pH	Extra compound	Protocol

0.03	12	3.5	Acetate (Ace)	B
0.05	30	5	Benzoate (Benz)	A
0.1		6.5	CO_2_	
0.2			Ethanol 18.72 mM (Eth)	
			Ethanol 9.38 mM (Eth)	
			Formate (For)	
			Propionate (Pro)	
			Sorbate (Sor)	
			Tween 80 (Twe)	
			none	

This table presents the different settings within each of the ten cultivation parameters. Each of the 55 conditions in the chemostat compendium is characterized by a combination of settings of the ten cultivation parameters. The colored matrix in Figure 1 is a schematic representation of the settings of the cultivation parameters for each condition. Abbreviations of cultivation parameter settings used in the schematic representation are stated between parentheses in this table.

**Figure 2:**
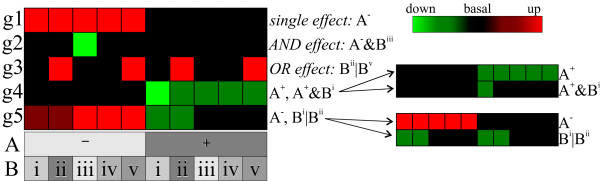
**Schematic representation of the normalized expression patterns of genes affected by a single effect, combinatorial effect or a mixture of these**. In this example there are two cultivation parameters, A and B, which can assume two and five different values, respectively. Genes g1, g2 and g3 are affected by a single effect, AND effect and OR effect, respectively. The expression of genes g4 and g5 is constituted by the influence of both a single effect and a combinatorial effect.

Here, the cultivation parameters form the predictors. We incorporated single effects and two types of combinatorial effects. See Figure 2 for a schematic example of genes that are influenced by these effects. A single effect is constituted by one setting of one cultivation parameter. For example, limiting element carbon is a predictor. (This will be a significant predictor for genes, which show differential expression between carbon-limited growth and growth that is limited by the residual quantity of other substrates.) In Figure 2 gene g1 responds solely to a single effect. The first type of combinatorial effect is constituted by applying the logic AND function between two settings of two different cultivation parameters. For example, limiting element carbon AND aerobic growth (in short: aerobic carbon-limited growth) form such a combinatorial effect. Of course, the cell's transcriptome and metabolome are known to respond in a combinatorial fashion to particular environmental conditions or parameters. That is, the simultaneous presence of certain environmental factors results in a transcriptional and metabolic state that is not a simple aggregation of the states reached based on the single presence of one of these factors. For example, when glucose is present, it is utilized in different ways by *S. cerevisiae*, depending on the presence of oxygen. Including these AND effects enables the systematic investigation of the influence of combinations of cultivation parameters on gene expression. Gene g2 in Figure 2 responds to an AND effect. The second type of combinatorial effect is constituted by applying the logic OR function on two different settings within the same cultivation parameter. Here, carbon-limited OR iron-limited growth forms an example.

This effect is included, because we expect that closely related settings within a cultivation parameter, e.g. similar carbon sources, will have a similar effect on gene expression. Gene g3 in Figure 2 responds to an OR effect. In the case of *UPC2 *(Figure 1), the regression model successively selected the single effect aeration type, the AND combinatorial effect anaerobic zinc-limited growth and the OR combinatorial effect nitrogen source proline or asparagine. (Note that cultivation parameter aeration type can assume only two settings, i.e. aerobic growth and anaerobic growth. Since these two predictors are mutually redundant, only one of them (aerobic growth) is included as a predictor in the regression model and labeled as aeration type. A positive regression coefficient for aeration type indicates that the gene is more highly expressed under aerobic conditions; a negative coefficient indicates the reverse scenario.) The regression model keeps on adding cultivation parameters as predictors, until no further significant improvement can be made. For example, for g4 in Figure 2 the single effect A^+ ^is selected first, followed by the combinatorial effect A^+^&B^i^. See Methods section for details.

### The expression of many genes responds to combinatorial effects

**Figure 3:**
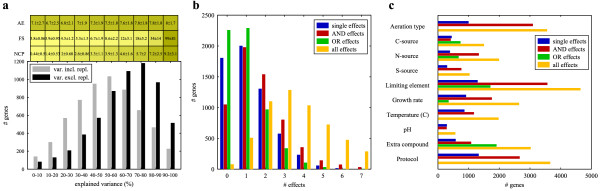
**General statistics of the applied regression model**. **a: **Histogram plot indicating how much variance within the gene expression patterns could be explained by the regression model for all (differentially expressed) genes. The black bars indicate the percentage of explained variance when excluding the variance present in the replicates, and which, therefore, cannot be explained by the regression model. Above the histogram are the mean and variance of the average expression level (AE), the F-statistic (FS) and the number of selected cultivation parameters (NCP) for the groups of genes with explained variance (including replicate variance) as stated on the x-axis of the histogram. **b: **Histogram plot indicating the number of single and combinatorial effects as well as the total number of effects that were selected to explain the observed gene expression patterns. **c: **Histogram plot indicating the number of genes influenced by particular cultivation parameters, either as a single effect, AND effect, OR effect or independent of the effect type ('all effects'). The 'all effects' bar is not the sum of the other three, because genes can be affected by a cultivation parameter both as a single effect and as a combinatorial effect.

For most genes the regression model was able to explain 60 to 80% of the variance, which is present in their expression patterns across the 55 conditions. See Figure 3a. The amount of explained variance does not depend that much on the average expression level of a gene, although there is a steady increase in explained variance with increasing average expression level. Much more important is the degree to which a gene is differentially expressed. The F-statistic, i.e. the ratio between the variance of the average expression levels across the 55 conditions and the average replicate variance across these conditions, is strongly correlated with the degree to which the gene's expression pattern can be reconstructed. The expression levels of genes with small F-statistics are obscured by measurement noise and do not differ significantly between the growth conditions. Also not surprisingly, when more significant cultivation parameters are selected by the regression model, more of the variance of the gene can be explained. Figure 3b, c outlines which and how many cultivation parameters were selected to reconstruct the expression patterns of all genes. On average, a gene is influenced by 1.25 (± 1.18) single effects, 1.73 (± 1.43) AND effects and 1.01 (± 1.04) OR effects. The limiting element, aeration type and protocol (which is dealt with in more detail below) are the most prominent factors that influence gene expression behavior. Here it should be noted that the setup of the cultivation parameters in the compendium is not fully combinatorial, i.e. not all possible combinations of cultivation parameters are present in the dataset. For example, across the 55 conditions, 53 have been cultivated under pH 5, while only a single condition was performed with a lower pH (3.5) and similarly for a higher pH (6.5), thereby precluding combinatorial effects between the higher or lower pH and other environmental parameters. Thus, the numbers of genes, which are influenced by a particular cultivation parameter (as visualized in Figure 3c), are biased by the number of different settings of the cultivation parameters and the number of combinations of cultivation parameters present in the compendium. Anyhow, the results indicate that the expression of many genes is influenced, not only independently by particular cultivation parameters, but also in a combinatorial fashion, i.e. there are many combinatorial effects between cultivation parameters that affect gene expression behavior.

**Figure 4:**
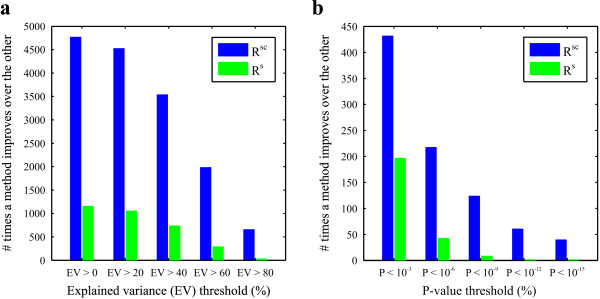
**Comparison between the regression analysis including including both the single and the combinatorial effects (R^sc^) and the regression analysis including only the single effects (R^s^)**. **a: **Histogram plot indicating how many times one method (R^sc ^or R^s^) leads to a higher percentage of explained variance (EV) of a gene given that the EV of this gene is larger than the EV threshold (x-axis) for at least one of both methods. **b: **Histogram plot indicating how many times one method (R^sc ^or R^s^) leads to a higher enrichment value (lower p-value) for a functional category given that the enrichment of this category is below a p-value threshold (x-axis) for at least one of both methods.

The regression analysis was repeated using only the single effects as predictors. For most genes this resulted in a lower percentage of explained variance. See Figure 4a. Of course, this result could be expected based on the fact that many combinatorial effects were selected as significant predictors in the original regression model. Subsequent enrichment analysis provided additional evidence for combinatorial regulation. Genes, of which their expression levels are manipulated by a particular single effect or combinatorial effect, were grouped and checked for functional overrepresentation. Additional File 1 provides an overview of all enrichment analysis results. It reveals the many cases (> 1000) in which a particular combination of environmental parameters leads to the up- or downregulation of a group of functionally related genes. Also, functional enrichment was compared between the regression analysis including both single and combinatorial effects and the analysis including only single effects. Genes were clustered based on their reconstructed expression patterns that were obtained for both regression models and these clusters were evaluated for enrichment in functional annotation categories. Figure 4b shows that the inclusion of the combinatorial effects leads to increased functional enrichment, and thus further substantiates the existence of the combinatorial influence of the presence of environmental factors and the importance of modeling them. Additional File 2 describes the complete comparison between the regression models including and excluding the combinatorial effects.

### The sample preparation protocol has a large impact on the measured gene expression levels

As indicated in Table 1 and Figure 1 the tenth cultivation parameter is termed "Protocol". Unlike the nine other parameters, "Protocol" is not directly related to the cultivation conditions under which yeast is grown, but refers to the protocol to process RNA samples. Several years ago, an improved sample preparation kit was introduced [14]. This kit obviated the need for the expensive and time-consuming poly-A mRNA purification step included in the original procedure. The decision to omit the purification step, which was also made in other yeast research groups, was supported by information indicating that samples prepared with or without this step were similar [15]. Thus, two different protocols were used to generate the chemostat compendium's samples for microarray hybridization: Protocol A and Protocol B. The main difference between these protocols is that Protocol A includes the polyA-mRNA isolation step (with cDNA synthesis being performed on purified mRNA), while Protocol B excludes the purification step (with cDNA synthesis being performed on total RNA). (The Methods section and Additional File 3 provide the complete details on both protocols.)

**Figure 5:**
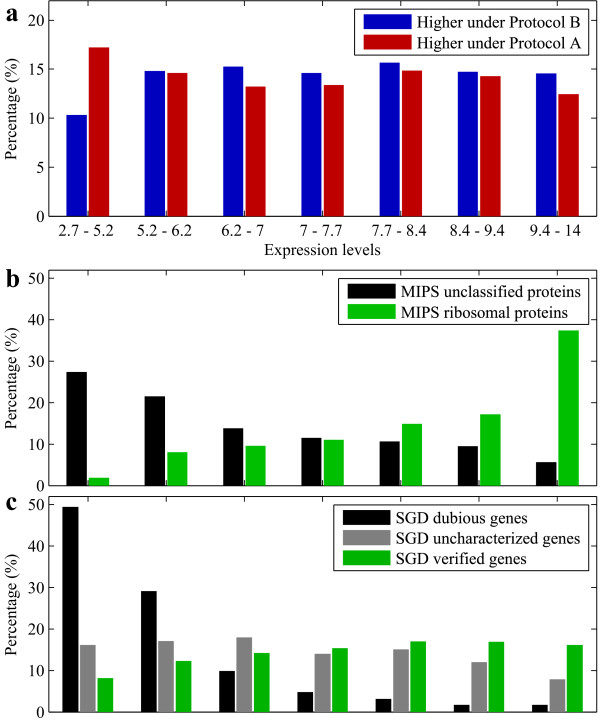
**The influence of the protocol on gene expression**. All genes that are affected by the modifications to the protocol, either as a single effect or as an interaction effect, are analyzed. First, the mean expression levels of these genes across all 55 conditions are computed. The genes are divived in seven groups based on their mean expression levels such that each group holds the same amount (i.e. 14,3%) of the genes. Each group is characterized by a lower and a higher bound on the expression value; these two numbers represent the range of the mean expression levels of the genes within the group. Also, we dichotomize the genes into the ones with positive regression weights (i.e. upregulation under Protocol B with respect to Protocol A) and the ones with negative regression weights. **a: **The blue bars indicate the percentage of genes with positive regression weights (higher under Protocol B) across these groups (or expression ranges). Similarly, the red bars indicate these percentages for the genes with negative coefficients (higher under Protocol A). **b, c: **For the same ranges, each bar represents the percentage of genes in the range annotated to a particular functional category over all of the genes that are annotated with this category and affected by the protocol.

As apparent from Figure 3c, the measured transcript levels of many genes appeared to be influenced by the protocol. Enrichment analysis revealed a significant overrepresentation of characterized genes amongst the genes that have higher apparent transcript levels under protocol B; all three GO root-categories (biological process, cellular component and molecular function) were highly enriched. On the other hand, significantly many uncharacterized genes yielded higher apparent transcript levels under protocol A. Further investigation revealed a trend between transcript level and protocol influence: Genes with higher average expression level tended to yield a higher transcript level in protocol B and genes with a lower average transcript level tended to yield lower transcript levels under protocol B (Figure 5). In general, uncharacterized genes have a lower expression than characterized genes, which explains the results from the enrichment analysis. Further evidence for this hypothesis is found when analyzing the genes that encode ribosomal proteins (RP genes), whose mRNA's are highly abundant. Again, significantly many RP genes exhibit higher expression when analyzed with protocol B (middle and bottom plots in Figure 5).

The relationship between mRNA abundance (expression level) and protocol is only weak and does not hold for each gene individually. It may, for example, be influenced by the average length of the polyA-tail of different transcripts. Indeed, analysis of mitochondrial genes lacking a poly-A tail demonstrated a large influence of the protocol. Of the 52 transcripts on the microarray representing mitochondrial genes, 27 (amongst which 16 unique mitochondrial genes) were influenced by the protocol, i.e. the regression model selected the protocol as a significant predictor of the expression pattern of these genes. All these 27 mitochondrial genes showed a higher (apparent) transcript level under protocol B. These results illustrate that not only different microarray platforms, labs, and strains, but also the hybridization preparation steps can affect the outcome of microarray analyses. This strongly underlines previous warnings on the challenges involved in comparing microarray results from different experiments.

The chemostat compendium allows us to adequately model the influence of the hybridization protocol on expression. In particular, the compendium contains 18 growth conditions (9 sets of two), where the only differing cultivation parameter is the protocol setting: The growth conditions were identical in these nine cases, only the protocol was different. This provides extra statistical power in the regression procedure and enables us to successfully model the protocol effect. This allows us analyze the influence of the environmental cultivation parameters without interference of the protocol's confounding effect.

### Functional categories are specifically associated with combinations of environmental parameters

Many functional categories are specifically influenced by a combinatorial effect. Many genes within such a category are influenced by a combinatorial effect, whereas none or only a few genes are affected by the single effects that constitute this combinatorial effect. See Methods section for these details. This analysis was performed on all MIPS categories. In total 153 significant combinatorial effect-MIPS category pairs were identified. These are depicted in Additional File 4. Here, we focus on the biological interpretation of two such combinatorial effects: Carbon source acetate OR ethanol, and, Limiting element phosphorus OR Sulfur. See Table 2.

**Table 2:** MIPS functional categories specifically associated with combinatorial effects

	Enrichment p-values			
MIPS category	single effects			comb. effect
	Acetate	Ethanol	both	Acetate | Ethanol

METABOLISM	0.065	0.077	1	7.8·10^-18^
metabolism of glutamate	1	0.048	1	1.4·10^-6^
C-compound and carbohydrate metabolism	0.027	0.082	1	1.4·10^-22^
C-compound and carbohydrate utilization	0.02	0.043	1	1.3·10^-17^
C-compound, carbohydrate catabolism	0.2	1	1	8·10^-13^
sugar, glucoside, polyol and carboxylate catabolism	0.44	1	1	9.3·10^-11^
ENERGY	0.013	1	1	1·10^-17^
glycolysis and gluconeogenesis	1	1	1	3.8·10^-9^
tricarboxylic-acid pathway	1	1	1	2.2·10^-11^

	Phosphorus	Sulfur	both	Phosphorus | Sulfur

transcriptional control	0.13	0.017	1	4.3·10^-8^
RNA processing	0.86	0.32	1	1.5·10^-6^
rRNA processing	0.5	0.83	1	3.3·10^-6^

The combinatorial effects 'carbon source acetate OR ethanol' and 'Limiting element phosphorus OR sulfur' are specifically associated to the listed MIPS functional categories. P-values of the enrichment of genes within these categories that are affected by the combinatorial effect are given in the rightmost column. Also, enrichment p-values of genes affected by each and by both of the single effects that constitute this combinatorial effect are given.

The first example is provided by the OR effect of carbon sources ethanol and acetate on metabolism and energy household. These C2-compounds share a drastically different impact on central metabolism when compared to using the sugars glucose, maltose and galactose as carbon source. During growth on sugars, all metabolic building blocks can be derived from glycolysis, the tricarboxylic acid cycle and the pentose phosphate pathway, while during growth on C2-compounds, gluconeogenesis and the glyoxylate cycle are essential for the provision of some of these precursors. Furthermore, the higher ATP requirement for biosynthesis during growth on the C2-compounds implies that, at a fixed specific growth rate, dissimilatory fluxes have to be higher with the C2-compounds than with a sugar as the sole carbon source. This is supported by the significant shared influence of the C2 carbon sources on the genes of gluconeogenesis and the tricarboxylic acid pathway.

Besides this and other examples that can be easily explained by current knowledge, there are also many interactions that might represent as of yet unknown regulatory mechanisms. For example, we find that the limiting elements sulfur and phosphorus have a similar effect (i.e. OR effect) on transcription regulation genes. A close inspection of the genes influenced by this OR effect revealed the presence of five genes encoding subunits of Mediator (*MED3*/*PGD1 *(complex tail), *MED7 *and *MED10*/*NUT2 *(middle), *MED11 *and *MED18*/*SRB5 *(head)), an evolutionarily conserved coregulator of RNA polymerase II [16] and nine genes encoding chromatin remodeling enzymes (*ARP7*, *GCN5*, *HST2*, *RIF1*, *RSC6*, *RVB2*, *SFH1*, *SNF6 *and *SPT8*). In eukaryotes, gene transcriptional regulation depends on a complex interplay between signal transduction, specific and general gene regulators and complexes that modify chromatin and RNA polymerase II. Under sulfur limitation *S. cerevisiae *adapts its transcriptome in order to reduce the expression of sulfur rich genes and proteins [17,18]. This response is mediated by Met4 the main sulfur metabolism regulator. The transcriptional changes upon phosphate limitation are mainly related to high affinity phosphate transport, phosphate assimilation and polyphosphate metabolism [5,18]. Although *S. cerevisiae *requires the transcription of different specific genes under sulfur or phosphate limitations, it is tempting to speculate that the mechanisms that govern the transcription control of these specific sets of genes are shared and depend on shared mechanisms involving specific subunits of the Mediator complex. Such high degree of specificity was demonstrated with the implication of Med2 (a Mediator tail subunit) in the regulation of the low iron response regulon [16].

### Combinatorial regulation within biochemical pathways provides further insight into sulfur metabolism and scavenging

As demonstrated above, we can assess whether groups of genes are influenced by particular (combinations of) environmental parameters using enrichment tests. This opens up the interesting possibility to correlate new and previously known patterns of regulation of individual genes with the regulation of larger families of genes connected to each other in pathways. In contrast to other gene groups, in a metabolic pathway clear connections exist between the gene products and their functions, which allows for more in-depth analysis. Here, we focus on biochemical pathways as described in SGD, which depict the series of chemical reactions converting metabolites, and the enzymes catalyzing these reactions. Enrichment analysis indicated that 5 of the 9 downloaded 'SGD superpathways' were influenced by at least one significant combinatorial effect (at *p *< 10^-3^, *q *< 0.08).

**Figure 6:**
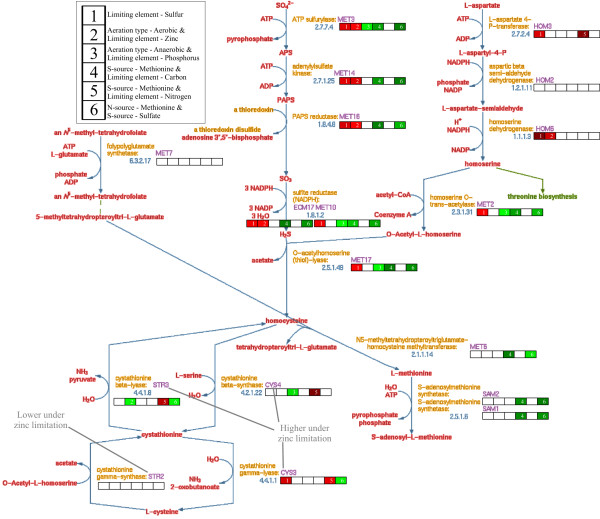
**Superpathway of sulfur amino acid biosynthesis**. Near each enzyme (gene product) is a bar representing the regression weights of the six significant cultivation parameters. These parameters are stated in the legend in the upper-left corner of this figure. A blank box indicates that the cultivation parameter is not selected by the regression model. Red and green boxes indicate positive (upregulation) and negative (downregulation) regression weights, respectively. Darker colors indicate larger regression weights.

**Figure 7:**
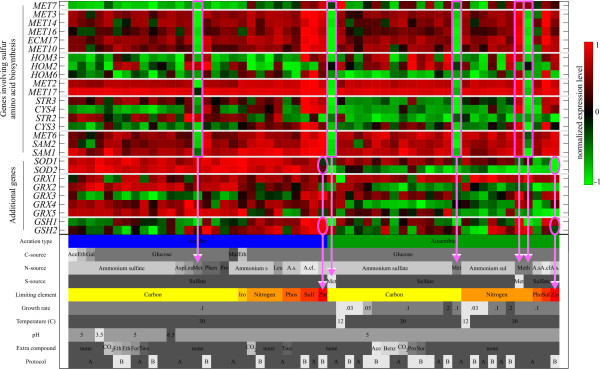
**Normalized gene expression patterns of the genes that are part of the superpathway of sulfur amino acid biosynthesis and additional genes discussed in the text**. The expression values of each gene are linearly scaled to range from -1 to 1. Here, -1 represents the lowest expression value and 1 indicates a gene's highest expression value. These normalized expression patterns are projected on the green-black-red colormap to derive the heatmap visualization. Separate branches of the pathway are indicated by the grey horizontal lines. For the group denoted as "Additional genes", the grey horizontal lines split the genes in functionally related groups. The magenta boxes and arrows indicate the cultivation parameters, where methionine is used as nitrogen or sulfur source. The magenta ellipses and arrows highlight the expression levels of the *SOD *and *GSH *genes under zinc limitation.

An illustrative example is presented by analyzing the expression profiles of the gene family involved in sulfur- and sulfur containing amino acid-metabolism in yeast (Figure 6). Sulfur amino acid biosynthesis involves a considerable number of enzymes required for the de novo biosynthesis of methionine and cysteine and the recycling of organic sulfur metabolites. Expression of the genes encoding the enzymes for this metabolic network is tightly controlled by the available sulfur source, through modulation of the intracellular S-adenosyl-methionine levels. Six different cultivation parameters were significantly often selected to explain the expression patterns of the genes in this pathway (*p *< 10^-3^). Five of these are combinatorial cultivation parameters. Not surprisingly, the only single effect is sulfur limitation, which causes the upregulation of ten out of the eighteen genes [19]. See box 1 of the bars near the enzyme names in Figure 6. Despite large variations in expression under different combinations of conditions, many of the *MET*-, *CYS*-, *SAM*- and *HOM *-genes invariably respond to the presence of methionine in the growth medium by clearly reduced expression. See Figure 7, which depicts the normalized gene expression patterns of all genes of the pathway. This response is independent of the presence of oxygen or growth limitation by carbon or nitrogen sources. Only in the case where methionine is utilized both as sulfur and nitrogen source *and *methionine is the limiting element, we observe that the expression of the corresponding genes is not reduced (but even slightly induced, mimicking the (known) response under sulfur limitation). This explains the selection of the combinatorial effects involving methionine depicted by boxes 4, 5 and 6 in Figure 6.

Interestingly, two genes involved in this sulfur-metabolizing network in part respond differently. *HOM2*, which is involved in homoserine biosynthesis, responds reciprocally to the availability of methionine in the growth medium compared to the other *HOM *genes, especially under aerobic conditions. The same observation is made for *STR2*, which is involved in cystathionine biosynthesis. (In Figure 7 magenta boxes mark the conditions, where methionine is part of the growth medium.) This discrepancy is indicative of a differential regulatory mechanism operating between the *HOM2*, *HOM3 *and *HOM6 *genes of the homoserine pathway, and of the complex regulation of the transsulfuration pathway, involving *CYS3*, *CYS4*, *STR2 *and *STR3*. Further detailed analysis would be required to elucidate the molecular mechanisms operating in these differential combinatorial controls. Such differential controls operating within a pathway are likely to be involved in intricate flux balancing mechanisms.

Surprisingly, for many of the genes in the pathway under investigation expression levels under zinc limitation are almost as high as under sulfur limitation, especially under aerobic conditions. Moreover, the genes of the transsulfuration pathway are highly expressed under zinc and sulfur limitation, yet lower expressed under the other nutrient limitations. Also here, *STR2 *responds reciprocally and is lower expressed under zinc limitation. Although transcript levels per se cannot be used as flux indicators, this expression behavior is consistent with an upregulation of the flux towards cysteine under zinc limitation via the increased synthesis of the corresponding enzymes. (See the graph structure of the pathway near cysteine in Figure 6.) The exact nature of this response is not immediately apparent. However, it provides an interesting hypothesis on the oxidative stress response of *S. cerevisiae *under zinc limitation. As previously described [20], a "first line of defense" in oxidative stress response is formed by the superoxide dismutase genes *SOD1 *and *SOD2*, which are induced under aerobic conditions. See Figure 7. The dithiol glutaredoxin genes *GRX1 *and *GRX2 *[21], and the monothiol glutaredoxin genes *GRX3 *-*GRX5 *[22], which also participate in the response against oxidative stress, exhibit highly differential transcriptional profiles.

This may provide new insight into the specific roles for each of the varying combinations of glutaredoxins under different growth conditions. Surprisingly, under zinc limitation not only the Cu, Zn-dependent *SOD1 *gene is lower expressed; also the *SOD2 *gene, encoding the mitochondrial superoxide dismutase, which is dependent on Mn and not on Zn, is much less induced. A boost in glutathione synthesis apparently takes over the main defense, since the glutathione synthase genes *GSH1 *and *GSH2 *are clearly induced, especially under zinc-limited aerobic conditions. This can be seen from the magenta ellipses in Figure 7. This fits with the fact that significantly many genes in the sulfur scavenging pathway are upregulated under zinc-limited aerobic growth, presumably leading to an induced cysteine pool, cysteine being one of the three components of the tripeptide glutathione.

### Functional characterization of uncharacterized and dubious genes using the chemostat compendium

In a recent review [23] it was pointed out that many (> 1000) genes in the yeast genome are still uncharacterized. Possible reasons for this include genetic redundancy, lack of strong growth phenotype and the possibility that not all of them are real genes. Additionally, genes may be involved in environmental and metabolic responses, which are normally not queried in the lab. Concerning the "characterized" genes, it can be noted that the function of many annotated genes is derived from large-scale studies, and hence, in-depth detailed analysis is lacking for these genes.

**Figure 8:**
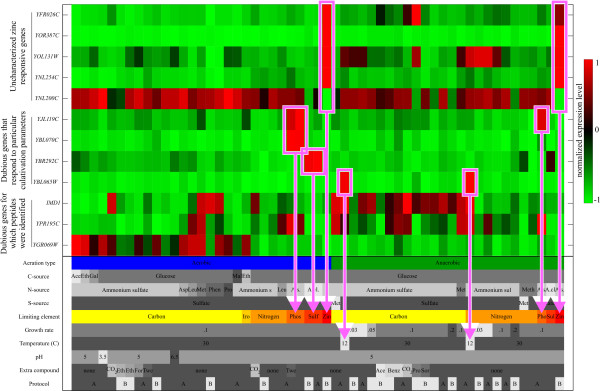
**Normalized gene expression patterns for twelve uncharacterized or dubious genes**. The expression values of each gene are linearly scaled to range from -1 to 1. Here, -1 represents the lowest expression value and 1 indicates a gene's highest expression value. These normalized expression patterns are projected on the green-black-red colormap to derive the heatmap visualization. The magenta boxes and lines highlight the cultivation parameters that influence the expression of the genes.

We conjecture that the visualization of the expression behavior of a gene over the conditions of the compendium, together with the identification of the significant cultivation parameters to which the gene responds, provides valuable information regarding gene function. With this information, one can design directed biological experiments or assays that probe a specific pathway or activity in order to advance towards the functional characterization of a gene. We mapped our regression results to SGD's genome snapshot, upon which the division of *Saccharomyces cerevisiae *ORF's into verified ORF's, uncharacterized ORF'S and dubious ORF's in [23] was based. For 1350 genes the regression model lead to a good reconstruction of the observed expression pattern (explained variance including replicate variance > 70%). According to SGD, 1009 of these genes were verified ORF's; 286 were uncharacterized and 54 were classified as dubious genes. Amongst the uncharacterized genes, many genes were found to be expressed under conditions which have not been extensively studied before. For example, amongst the 286 uncharacterized genes, five genes are most significantly influenced by zinc limitation, i.e. zinc limitation was the first condition selected by the regression model. One of these, *YOR387C*, is only expressed under zinc limitation. These results immediately link the function of a gene to a particular cultivation parameter or a specific biological process related to this cultivation parameter. The expression pattern of these five zinc responsive genes as well as the other genes to be discussed in this section are visualized in Figure 8. Also, amongst the 54 dubious genes, there are many genes that are highly expressed under one or a few cultivation parameters, while having a constant expression over the remaining conditions. For example, *YJL119C *is only highly expressed under phosphorus limitation. *YBL070C *also responds to phosphorus limitation, yet particularly when the yeast is grown aerobically. The expression of *YBR292C *is influenced by aerobic sulfur-limited growth and *YBL065W *is only expressed when grown at a low temperature (12°C). 35 of the 54 dubious genes were affected by the aeration effect or the interaction effect between carbon limitation and aeration. These genes were screened against a recent proteomics study, where expression data of yeast grown in aerobic and anaerobic carbon-limited chemostats was measured [24]. We found that for three genes unique peptides were quantified. This establishes the existence of the proteins encoded by these "dubious" genes. See Additional File 5 for a list of the 54 dubious genes and details on the peptide identification. Notably, 51 of the 54 dubious genes are no longer present on YG 2.0, the successor of the Affymetrix YG S98 GeneChip, after comparative genomics [25] and phylogentic footprinting [26] approaches identified these as false ORF's. However, our analysis reveals a clear-cut influence of environmental conditions on the expression levels of many of these genes, implying that these genes *do *have a functional role, at least in the *Saccharomyces cerevisiae *strain that was used in this study.

### Analysis of shake-flask experiments with the chemostat compendium

Changes in the extracellular environment or perturbations on genetic level do not only affect (signaling) pathways in which the change or perturbation has direct involvement, but can also impact the cell's viability, metabolism or other processes in the cell. For example, there are many experimental conditions and genetic perturbations that will impact the growth rate of the cell. For shake flask cultivations it is not possible to distinguish between the direct and indirect effects, since cultivation parameters like growth rate and nutrient availability cannot be controlled. This also confounds the analysis of gene expression data from shake flask experiments [11]. By screening a group of genes, which were grouped together on the basis of shake flask experiments, against the compendium, some of the confounding effects can be resolved. The group can be subdivided into clusters of genes that respond to particular environmental parameters within the compendium and thereby identify the cultivation parameters or biological processes that could have played a role in the original shake flask experiment, even when these have not been measured.

**Figure 9:**
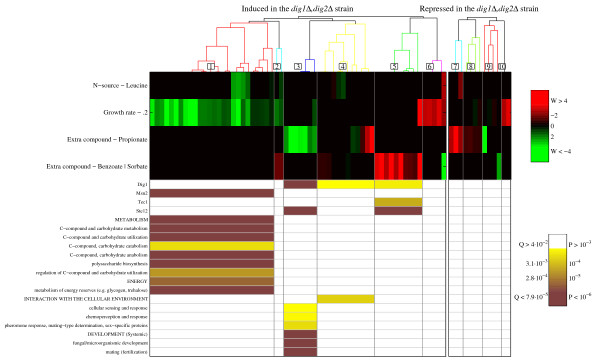
**Analysis of two groups: The genes upregulated in a *dig1*Δ, *dig2*Δ strain and the genes downregulated in this strain**. **Middle: **Normalized regression weights for the significant cultivation parameters across the gene groups. **Top: **The genes were clustered based on these regression weights. **Bottom: **Schematic representation of the enrichment p-values and related false discovery rates (q-values) for each of the uncovered clusters when related to TF binding data and MIPS functional categories.

To this end, we apply the following strategy: First, we select the (combinatorial) cultivation parameters that are significant for the group under investigation. These are the cultivation parameters that are significantly often selected by the regression model to explain the expression pattern of the genes in the group when compared to the complete genome. Next, the genes are clustered based on the normalized regression coefficients under these cultivation parameters. Finally, these newly obtained clusters are consulted for enrichment of annotation categories. See Methods section for details. As an example, Figure 9 depicts the results of this analysis for the groups of genes, which were found to be induced or repressed in a *dig1Δ*, *dig2Δ *mutant strain grown in a shake-flask [27]. To make the induced and the repressed gene groups, we consulted the gene expression data of this study (i.e. the Hughes *et al*. yeast mutant microarray compendium [27]). The induced group is formed by all genes that are upregulated by one fold-change or more in the *dig1Δ*, *dig2Δ *mutant strain compared to the wild-type strain. The repressed group is formed in a similar fashion by identifying the genes that are downregulated by one fold-change or more.

The results show a clear difference between direct and indirect effects. On the one hand, the enrichment analysis on the TF binding data tells us that the genes in Clusters 3, 4 and 5 form a significantly large part of Dig1's regulon, i.e. the direct targets of TF Dig1. The known role of Dig1 and Dig2 in regulating mating-specific and pheromone-responsive genes is confirmed by the enrichment of these functional categories in Cluster 3. Also, binding sites of TFs Tec1 and Ste12, which together with Dig1 form a regulatory complex involved in mating and filamentation [28], are enriched for Cluster 5 and Clusters 3 and 5, respectively. Interestingly, the genes within Clusters 3, 4 and 5 were clustered together based on their response to the addition of organic acids propionate, benzoate and sorbate. (The clusters are characterized by the shared transcriptional response of their genes to these acids.) On the other hand, a large set of genes that is induced after the knockout of *DIG1 *and functionally redundant *DIG2*, is affected by growth rate in the chemostat microarray compendium. See Clusters 1, 6 and 10. The genes of Cluster 1 show high enrichment for metabolism and energy functional categories as well as for general stress response TF Msn2. From this observation we conclude that besides the genes that are directly affected, the double knockout also has a large impact on the metabolism and energy household of the cell when grown in a shake-flask.

## Conclusion

The compendium of chemostat-based transcriptome data is a valuable resource for yeast systems biology that can be queried on-line. Additional File 6 contains the complete dataset (expression data and description of the cultivation conditions). Additional File 7 is an interactive tool to visualize the gene expression across all conditions in the compendium; this file can be downloaded from the author's website. Using a forward step-wise regression strategy, we were able to quantify the influence of (combinatorial) cultivation parameters on the expression of genes and (using enrichment tests) groups of functionally related genes. The regression results demonstrate the large extent to which regulation of individual genes results from the integration of multiple external signals. In fact, the analysis yielded only few "signature transcripts", i.e. transcripts whose level showed a unique up- or downregulation under a single condition in the compendium relative to all other conditions. This observation has important implications for the applicability of so-called signature transcripts to diagnose cellular status (e.g. starvation for a nutrient, stress or, in higher organisms, disease). Our results indicate that the "signature" status of a gene with respect to an individual environmental parameter can depend strongly on other ("background") environmental signals to which the cell is exposed. In this respect, it should be stressed that the current compendium of chemostat-based data represents only a minute fraction of the infinite range of combinatorial conditions to which yeast cells can be exposed in nature, in industry and in the laboratory.

The relevance of the proposed approach for functional analysis of genes and pathways is exemplified by the observed combinatorial effects of zinc and sulfur availability in the pathway of sulfur amino acid biosynthesis. Furthermore, the compendium approach has provided clear indications that 54 *S. cerevisiae *genes that had previously been labeled as 'dubious' and have even been removed from some commercial DNA microarrays, exhibited a specific and reproducible transcriptional response to some of the investigated culture conditions. These examples illustrate the potential for enabling more focused functional analysis studies through a correlation of a wide range of cultivation conditions and gene expression data. The results provide a strong incentive for further extending the range of cultivation conditions included in the compendium.

The systematic dissection of the impact of (combinations of) individual culture parameters on transcriptional regulation enabled by chemostat-based microarray analysis can be applied to interpret transcriptome data generated in less extensively controlled, but highly relevant cultivation conditions in industry and in the laboratory. This is exemplified by the additional interpretation of previously published data from shake-flask-based transcriptome analysis of a *dig1Δ*, *dig2Δ *mutant (Figure 9).

In view of the excellent reproducibility of chemostat-based microarray analysis [29], it should be possible to extend the compendium with data from other research groups, provided that yeast strain, cultivation procedures and procedures for microarray analysis are rigorously standardized. The effect of a change in the mRNA processing protocol, as identified in the regression strategy, provides a clear caveat on the possible impact of even small differences in experimental procedures.

One promising avenue to be explored is the use of the compendium in deriving transcriptional regulation networks. Given that changes in gene expression can be ascribed to changes in the activity of TFs and chromatin remodeling proteins, the compendium dataset provides the means to investigate how cultivation parameters influence the activity of the proteins that control transcription. Since the cultivation parameters, such as the employed carbon source, are closely linked to the actual molecular signals that are detected by the cell, it may be possible to also relate transporters and signaling cascades to the observed expression under different environmental conditions. This allows for a genome-wide analysis of the complete chain of regulatory relationships that cause changes in the extracellular environment to lead to changes in gene expression.

In the employed regression model, the (combinatorial) cultivation parameters are assumed to have an additive effect on gene expression. In previous work [6] the aeration type was modeled as a linear effect with both additive and multiplicative components. This approach was not possible for the cultivation parameters within the current framework. Furthermore, a more complex modeling or incorporation of higher-order effects results in a highly under-determined system and possible computational complexity issues. Given the high-degree of non-linearity in biological systems, the application of logic (Boolean) functions might provide a sensible alternative to the commonly used linear modeling. Irrespective of the structure of the models, incorporating combinatorial effects in models for (transcriptional) regulation is crucial. Only in this way, the goal of systems biology to investigate and understand the interactions between different components and/or levels in biological systems can be complemented by an equally integrative approach towards the complex environmental context in which cells grow and survive.

## Methods

### Chemostat cultivation and microarray data

Prototrophic *Saccharomyces cerevisiae *strain CEN.PK113-7D (*MAT*a) [30] was grown at 30°C (or at 12°C) in 2-liter chemostats (Applikon) with a working volume of 1.0 liter as described in van den Berg *et al*. [31]. Cultures were fed with a defined mineral medium that limited growth by either carbon, nitrogen, phosphorus, sulfur, zinc or iron with all other growth requirements in excess and at a constant residual concentration. The dilution rate ranged from 0.03 to 0.2 h^-1^. The pH was measured online and kept constant at 5.0 (or 3.5 and 6.5) by the automatic addition of 2 M KOH using an Applikon ADI 1030 bio controller. Stirrer speed was 800 rpm, and the airflow was 500 ml min^-1^. Dissolved oxygen tension was measured online with an Ingold model 34-100-3002 probe and was above 50% of air saturation. The off-gas was cooled by a condenser connected to a cryostat set at 2°C, and oxygen and carbon dioxide were measured offline with an ADC 7000 gas analyzer. When required, anaerobic conditions were maintained by sparging the medium reservoir and the fermentor with pure nitrogen gas (500 ml min^-1^). Furthermore, Norprene tubing and butyl septa were used to minimize oxygen diffusion into the anaerobic cultures [32].

Steady-state samples were taken after ~10–14 volume changes to avoid strain adaptation due to long term cultivation [33]. Dry weight, metabolite, dissolved oxygen and gas profiles had to be constant over at least 3 volume changes before sampling for RNA extraction. The detailed culture media recipes, used in the 55 different conditions presented in this study, can be retrieved from the individual GEO [34] array reports. The GEO accession numbers can be found in Additional File 6.

In this study, two different sample preparation protocols were employed: Protocol A (for 36 of the 55 conditions) and Protocol B (for 19 of the 55). For Protocol A, sampling of the chemostat cultures, probe preparation and hybridization to the single-channel Affymetrix GeneChip YG S98 microarrays is described in Piper *et al*. [29]. Protocol B has the following modifications with respect to Protocol A: In stead of harvesting ~700 *μ*g of total RNA and applying a Poly-A mRNA isolation step before cDNA synthesis (Protocol A), ~15 *μ*g of total RNA is harvested and the purification step is omitted (Protocol B). Thus, in Protocol B cDNA synthesis is performed on total RNA, while in Protocol A the synthesis is performed on Poly-A purified mRNA. Additional File 3 provides the complete details on both protocols and references to the used AffyMetrix manuals.

Across the 55 conditions, ten different varying cultivation parameters can be identified. A cultivation parameter, e.g. the carbon source, is described as a categorical variable and contains two or more settings, e.g. the used carbon source can be either acetate, ethanol, galactose, glucose or maltose. Each condition is characterized by a configuration of these settings across the ten cultivation parameters. See Figure 1, Table 1 and Additional File 6 for an overview of the relevant settings within the environmental parameters per condition. In total, 180 microarray measurements were performed. There is a variable number of independent biological replicates per condition, however for most (39) conditions three replicates were performed. Chip quality control, condensing probe intensities to gene expression levels and normalization was performed using GeneData Refiner Array [35]. 170 high quality chips, i.e. gradient severity ≤ 0.165, defective area ≤ 0.5% and outlier area ≤ 0.59%, were retained. Ten chips, which did not meet these criteria were dismissed. The RMA algorithm was used to derive the log scale measure of the expression levels [36]. Quantile normalization was applied to normalize between arrays [37]. The normalized expression data is given in Additional File 6. The raw array data used in this study can be retrieved at Genome Expression Omnibus [34] with series number GSE11452.

### Detecting differential expression

A gene was called differentially expressed when 1) the gene was present in at least one of the arrays (present call p-value < 0.05) and 2) the gene showed significant differential expression in at least one condition (one-way ANOVA with 55 classes, p-value < 0.05/9335). 9335 is the total number of transcripts on the array.

### Inferring the influence of cultivation parameters on gene expression using regression

A designmatrix was created, containing both main (or single) effects and interaction (or combinatorial) effects: Each setting within each cultivation parameter is represented by a binary indicator column with 170 entries. These columns represent the main effects, which indicate for each array whether the yeast was grown under the relevant setting of a particular cultivation parameter. Two types of combinatorial effects were included in the model, i.e. "AND" and "OR" effects. The AND interaction effect columns were obtained by applying the logical AND function to all possible pair-wise combinations of main effect columns. The OR interaction effect columns were obtaining by applying the logical OR function to all possible pair-wise combinations of main effect columns that are associated with the same cultivation parameter. Thus, only OR effects that are constituted of two settings within the same cultivation parameter were modeled. Redundant columns and columns with all zeros were removed. This resulted in the binary [170 × 227] designmatrix **D**, which includes 38 single effects, 101 AND effects and 88 OR effects. A visualization of this matrix is found in Additional File 8.

A forward step-wise ordinary least squares regression strategy was applied to each gene individually:

**y **= **X***β *+ *ε*

Here, **y**_*i *_denotes the measured gene expression level of a particular gene for array *i*, with *i *= 1, . . ., 170; **X **is the predictor matrix, *β *represents the regression coefficients and *ε *the error, which is assumed to be independent zero-mean normally distributed. Initially, **X **contains only the intercept, i.e. a column of 170 ones. In an iterative fashion, columns from **D **are added to **X**. For this we applied a leave-one-out cross validation (loocv) scheme, where a single sample is used for testing, while the remaining (169) samples are used for training the regression model. This was repeated such that each sample is used once as the test data. The column from **D**, with the smallest root-mean-squared (rms) loocv error and absolute regression coefficient larger than 0.3, was selected and added. The iterative process of adding columns is discontinued when the p-value, as output by a t-test that determines whether the regression coefficient significantly differs from zero, exceeds 0.05/227. To prevent the inclusion of spurious AND effects, the following strategy is applied: When an AND effect column is selected, we check whether the addition in explained variance is larger than the addition is explained variance when adding the two main effect columns that constitute the combinatorial effect. Only in this case, we add the AND effect column, otherwise the two main effect columns are added, provided that they satisfy the p-value threshold and their absolute regression coefficients are larger than 0.3.

Note that only coefficients larger than 0.3 or smaller than -0.3 were allowed. This was done to focus on large changes in gene expression. Inclusion of absolute weights smaller than 0.3 did not increase enrichment scores of functional categories (see next section). Although small regression coefficients might be biologically relevant, this indicates that there are also many spurious results amongst the small regression weights.

The choice for a step-wise regression approach is substantiated in Additional File 9.

### Enrichment analysis

For each main or interaction effect, i.e. a column from **D**, we group all genes, for which that effect turned out to be a significant predictor with a positive regression coefficient (or regression weight). This procedure was also carried out to group genes with negative weights for the significant predictors, and to group genes irrespective of the sign of the weight. The latter grouping is basically a union of the genes with positive weights and the genes with the negative weights. In addition, for the cultivation parameters that can assume more than two settings, we group all the genes that respond to at least one of the settings of that cultivation parameter as a main effect. Basically, we select all main effect columns from **D **that represent a setting of one particular cultivation parameter and group the genes, for which at least one of these settings is a significant predictor. (Note that the cultivation parameters that can only assume two settings, i.e. Aeration type, S-source, Temperature and Protocol, only have one main effect column in **D**, since the two settings are mutually redundant and only one of them is included in **D**.) Also here, we make the distinction between positive and negative regression coefficients and the union of these. The hypergeometric test is employed to assess the significance of the overlap between all these groups and gene sets from GO [38], MIPS [39], KEGG [40] and TF binding data [41,42]. Additional File 1 provides an overview of the significant results (*p *< 10^-6^, *q *< 8.5·10^-4^). Here, for each triplet of p-values, associated with the positive weights, the negative weights or all weights, the most significant (smallest p-value) is selected and color coded accordingly. See page 1 of Additional File 10 for a flowchart describing the steps of this analysis.

### Functional categories specifically influenced by a combinatorial effect

To find a combinatorial effect that is specific for a functional category we group all the genes for which this effect was selected as a significant predictor by the regression model (irrespective of the sign of the weight). Also, for this effect, we make three other gene sets by grouping the genes which are influenced by 1) one of the single effects that constitute the combinatorial effect 2) the other single effect and 3) by both these single effects. (If a gene is influenced by both the combinatorial effect and a single effect, we only consider the effect that was selected first and then add this gene to the appropriate group.) Functional categories, which are overrepresented in the first group (*p *≤ 10^-5^) and not overrepresented in the three other groups (*p *≥ 10^-2^) are called "specifically influenced by the combinatorial effect". See page 2 of Additional File 10 for a flowchart describing the steps of this analysis.

### Clustering of genes based on regression coefficients

Given a group of genes, the hypergeometric test is employed to select those (interaction) effects, i.e. columns from **D**, which are significantly often selected by the regression model for the genes in this group when compared to all genes in the genome. Columns with *p *< 10^-5 ^are kept. Next, we create matrix **R**, which contains the normalized regression weights for the selected columns of all genes in the group under investigation. The normalized weights of a gene are obtained by dividing the original regression weights by the variance of the gene. A consensus clustering algorithm [43] is applied to cluster the genes based on the normalized regression weights in **R**: The data is clustered using a Bayes mixture of Gaussians EM algorithm. The number of clusters is varied from 2 to 20 (or the number of genes in the group if this is smaller than 20) and repeated 50 times for each number of clusters. The total of all clusterings is used to build a co-occurence matrix, which indicates how many times a pair of genes was found in the same cluster amongst all clusterings. This co-occurence matrix is transformed into a distance matrix. The distance matrix is zero, when a pair of genes was clustered together in all attempts; the matrix is one, when a pair never clustered together. We apply hierarchical clustering with complete linkage on this distance matrix and cut the dendrogram at 0.9 to create the final clusters. These clusters are consulted for enrichment of annotation categories using the hypergeometric test as explained before. See page 3 of Additional File 10 for a flowchart describing the steps of this analysis to create matrix **R**.

## Authors' contributions

TAK, JMD and MAB gathered and annotated the microarray data. TAK, MJTR and LFAW devised the methodology. TAK performed the computational experiments. TAK, JMD, JHW and JTP analyzed the data. TAK, JMD, JHW and JTP drafted the manuscript. PDL, MJTR and LFAW assisted in drafting and structuring the manuscript. All authors read and approved the final manuscript.

## Supplementary Material

Additional file 1 **Functional enrichment of genes influenced by the cultivation parameters.** This files gives, for each of the ten cultivation parameters, the functional enrichment of genes manipulated by one of the settings of a particular cultivation parameter, either as a single effect or as part of an interaction effect. Here, the first columns of the tables related to the cultivation parameters that can assume more than two settings, form an exception; these columns represent the enrichment of the genes that are manipulated by at least one of the settings of a particular cultivation parameter as a single effect. The genes in such a column are the union of the genes related to other single effect columns in the table. The (uncorrected) P-values and Q-values (FDRs) are represented by shades of red, green and blue. Here, red, green and blue refer to the enrichment of genes with only positive regression weights, only negative regression weights, or with all regression weights (independent of the sign), respectively. The consulted annotation data consists of GO biological processes (BP), cellular components (CC) and molecular function (MF), MIPS functional categories, KEGG pathways and TF binding data. Note that for GO we consider two types of data: One indicates whether a gene is assigned to a particular leaf in the GO annotation tree (annotated with appendix leaf). The other associates a gene located in a certain leaf not only with that particular leaf but also with all nodes between the leaf node and the root of the GO tree (annotated with appendix comp). Numbers next to the vertical axis indicate the group size of the functional catergories. Numbers beneath the horizontal axis indicate the number of genes in a gene group, i.e. the number of genes manipulated by a particular main or interaction effect of cultivation parameters. Numbers in the boxes indicate the overlap between these two groups. See Methods section – Enrichment analysis for details on how the gene groups, which respond to the cultivation parameters, were created. Click here for file

Additional file 2 **Comparison between regression analysis including and excluding combinatorial effects.** This file describes the comparison between the regression analysis including and excluding combinatorial effects by analyzing the explained variance and functional enrichment of clustered genes. Click here for file

Additional file 3 **Details of the different protocols.** This file states the details of the different protocols to prepare samples for hybridization to the microarray. References to the corresponding manuals are given. Also, advantages and disadvantages of both protocols are given. Click here for file

Additional file 4 **MIPS functional categories that are specifically influenced by combinatorial effects.** The significant category-effect-pairs are depicted by the dark boxes. The grey value of a box indicates the enrichment p-value and associated false discovery rate (q-value). On the right of the figure are the names of the significant MIPS categories; on the left is the hierarchy within these categories; the combinatorial effects are listed above the figure. The two cases that are discussed in the text are indicated by the magenta boxes. The corresponding MIPS categories and cultivation parameters are printed in bold and magenta. Click here for file

Additional file 5 **Peptide quantification and BLAST results for dubious genes.** This file provides information on the peptide quantification and BLAST results for three dubious genes. Also, it states which of the 54 dubious genes are not found on YG 2.0, the successor of the Affymetrix YG S98 GeneChip. Click here for file

Additional file 6 **Cultivation parameters across the compendium.** This file states the settings of the ten cultivation parameters for each of the 170 cultivations and subsequent microarray analysis. For each array, GEO sample and series numbers are given. For arrays that have been published along with previous studies the Pubmed IDs are given. The file also contains the normalized expression levels across the 170 microarrays for all genes. Click here for file

Additional file 7 **Interactive gene expression visualization tool.** This file contains the download link of a self extractable executable that contains an application that allows one to visualize the expression patterns of genes across the compendium. The conditions within the compendium can be sorted on two of the ten cultivation parameters. It is possible to analyze the significant cultivation parameters selected by the regression model to reconstruct the gene expression patterns. The Matlab code for this visualization tool can be obtained via the corresponding author. Click here for file

Additional file 8 **Visualization of designmatrix **D**.** This file is a visualization of the [170 × 227] binary designmatrix **D**. This matrix indicates for each of the 170 arrays/cultivations under which of the 227 (combinatorial) cultivation parameters the yeast was grown. These are marked by the non-white elements, which represent the 1's of **D**. (White elements represent the 0's.) For visibility, the transpose of the matrix is displayed. The x-axis contains the 170 array IDs; the y-axis displays the descriptions of the 227 predictors (which include 38 single effects (in red), 101 AND effects (in green) and 88 OR effects (in blue)). Click here for file

Additional file 9 **Choice of the regression strategy.** The choice for a step-wise regression approach is substantiated. Click here for file

Additional file 10 **Flowcharts of enrichment analysis.** Page 1 – Flowchart of Methods – Enrichment analysis. All significant regression weights are stored in a large matrix. This matrix has 6005 rows, which represent all differentially expressed genes for which the regression analysis was performed, and 227 columns, which represent the (combinatorial) cultivation parameters, which were used as predictors in the regression model. An element of this matrix indicates the assigned regression weight of a cultivation parameter to explain the expression pattern of a gene. A zero indicates that the cultivation parameter was not selected as a significant predictor for a gene. For each column of this matrix, we group the genes that were upregulated (positive weights), downregulated (negative weights) or either up- or downregulated (non-zero weights) by the cultivation parameter related to that column. These groups are analyzed for significant overlap with gene groups derived from functional annotation databases and TF binding information using the hypergeometric test. The enrichment results are visually represented in Additional File 1. Page 2 – Flowchart of Methods – Functional categories specifically influenced by a combinatorial effect Gene groups are derived based on the large matrix with regression weights. In this case, for each combinatorial effect we create four gene groups: 1) We group the genes that respond to the combinatorial effect (non-zero weights). 2) We group the genes that respond to one of the single effects that constitute the combinatorial effect. 3) We group the genes that respond to the other single effect. 4) We group genes that respond to both single effects. Again, the hypergeometric test is employed to analyze these gene groups for significant overlap with gene groups derived from functional categories. Page 3 – Flowchart of Methods – Clustering of genes based on regression coefficients Firstly, genes that respond (non-zero weights) to a particular (combinatorial) cultivation parameter are grouped. (These groups are identical to the blue groups generated in the enrichment analysis flowchart on Page 1 of this document.) These groups are analyzed for significant overlap with the gene group derived from the shake-flask experiment. The significant cultivation parameters are selected. (These are the cultivation parameters to which significantly many of the genes in the shake-flask gene group respond.) A new matrix is derived. This matrix contains the regression weights of the genes of the shake-flask gene group for the signficant cultivation parameters. Bascially, this matrix is a submatrix of the original regression weight matrix. By normalizing these regression weights, we arrive at matrix **R**, which is used in the subsequent cluster analysis. Click here for file
