# Supplementary material for: Combinatorial effects of environmental parameters on transcriptional regulation in Saccharomyces cerevisiae: A quantitative analysis of a compendium of chemostat-based transcriptome data
Source: BMC Genomics. 2009 Jan 27;10:53. doi: 10.1186/1471-2164-10-53 (PMC2640415; doi:10.1186/1471-2164-10-53)
Supplement: Additional file 1 — Functional enrichment of genes influenced by the cultivation parameters. This files gives, for each of the ten cultivation parameters, the functional enrichment of genes manipulated by one of the settings of a particular cultivation parameter, either as a single effect or as part of an interaction effect. Here, the first columns of the tables related to the cultivation parameters that can assume more than two settings, form an exception; these columns represent the enrichment of the genes that are manipulated by at least one of the settings of a particular cultivation parameter as a single effect. The genes in such a column are the union of the genes related to other single effect columns in the table. The (uncorrected) P-values and Q-values (FDRs) are represented by shades of red, green and blue. Here, red, green and blue refer to the enrichment of genes with only positive regression weights, only negative regression weights, or with all regression weights (independent of the sign), respectively. The consulted annotation data consists of GO biological processes (BP), cellular components (CC) and molecular function (MF), MIPS functional categories, KEGG pathways and TF binding data. Note that for GO we consider two types of data: One indicates whether a gene is assigned to a particular leaf in the GO annotation tree (annotated with appendix leaf). The other associates a gene located in a certain leaf not only with that particular leaf but also with all nodes between the leaf node and the root of the GO tree (annotated with appendix comp). Numbers next to the vertical axis indicate the group size of the functional catergories. Numbers beneath the horizontal axis indicate the number of genes in a gene group, i.e. the number of genes manipulated by a particular main or interaction effect of cultivation parameters. Numbers in the boxes indicate the overlap between these two groups. See Methods section – Enrichment analysis for details on how the gene groups, which respo [file 1471-2164-10-53-S1.pdf]

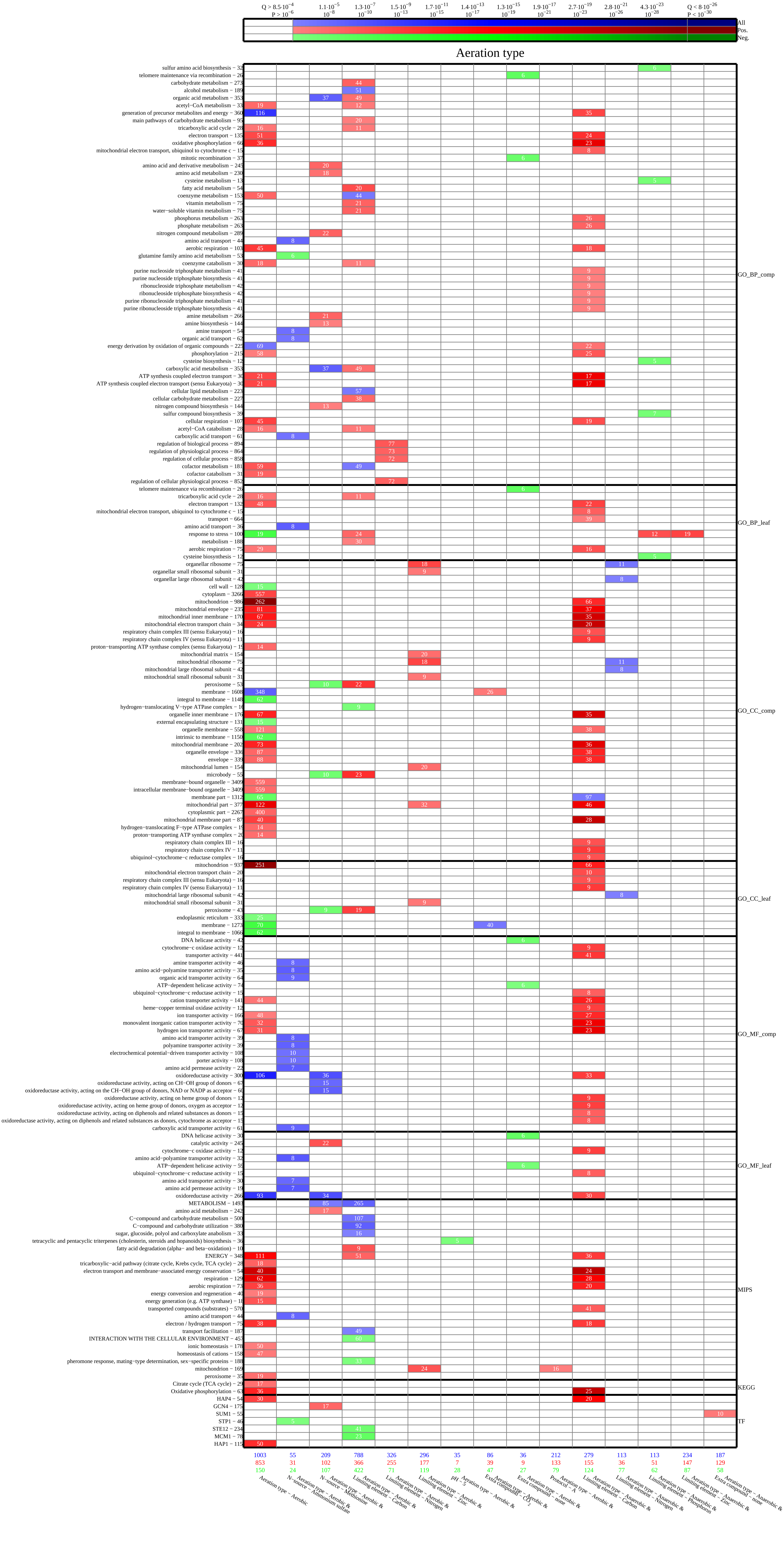

Aeration type
All
Pos.
Neg.
GO_BP_comp
GO_BP_leaf
GO_CC_comp
GO_CC_leaf
GO_MF_comp
GO_MF_leaf
MIPS
KEGG
TF

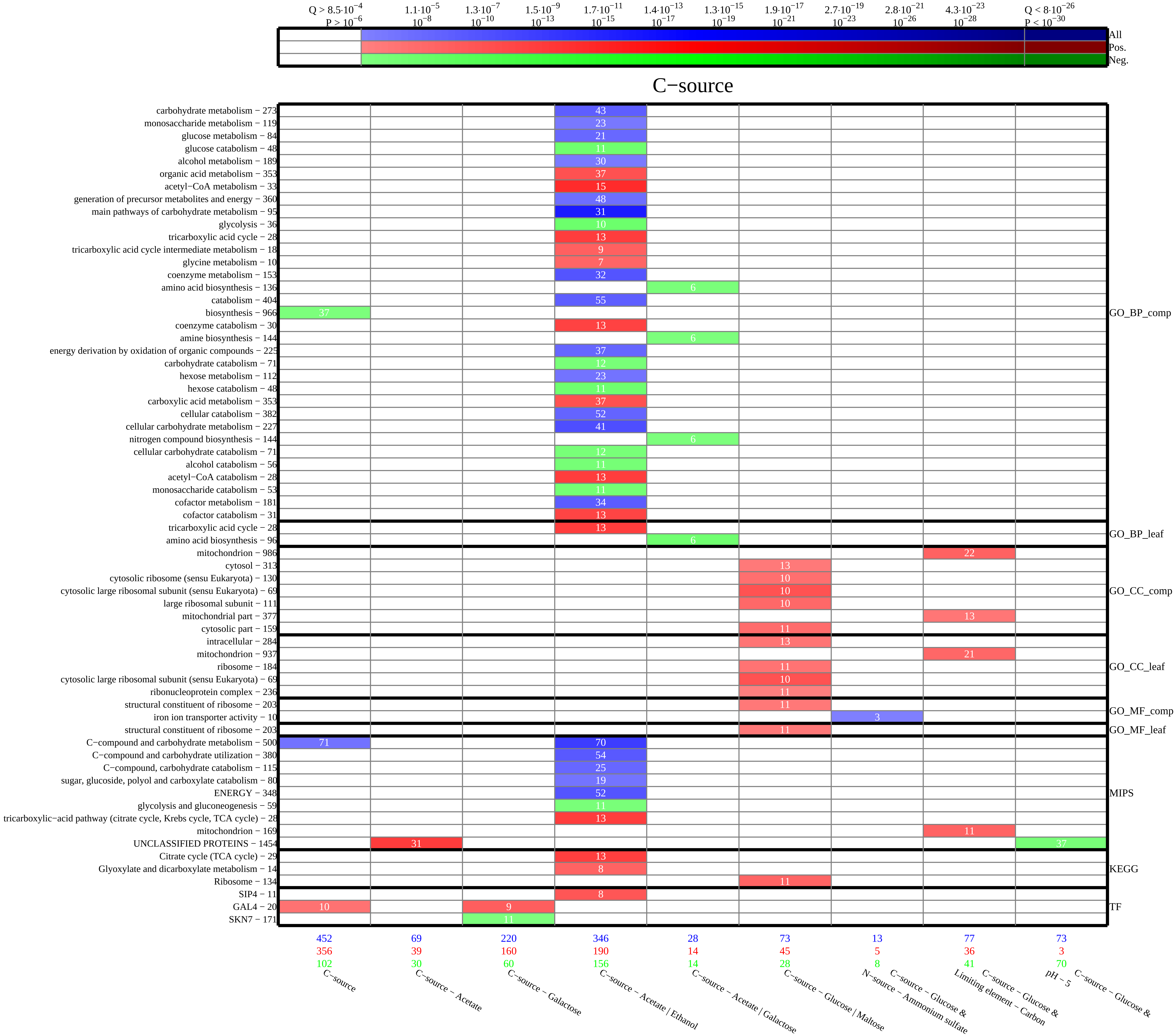
Q > 8.5·10^-4 P > 10^-6
1.1·10^-5 10^-8
1.3·10^-7 10^-10
1.5·10^-9 10^-13
1.7·10^-11 10^-15
1.4·10^-13 10^-17
1.3·10^-15 10^-19
1.9·10^-17 10^-21
2.7·10^-19 10^-23
2.8·10^-21 10^-26
4.3·10^-23 10^-28
Q < 8·10^-26 P < 10^-30
All
Pos.
Neg.
C−source
carbohydrate metabolism − 273 43
monosaccharide metabolism − 119 23
glucose metabolism − 84 21
glucose catabolism − 48 11
alcohol metabolism − 189 30
organic acid metabolism − 353 37
acetyl−CoA metabolism − 33 15
generation of precursor metabolites and energy − 360 48
main pathways of carbohydrate metabolism − 95 31
glycolysis − 36 10
tricarboxylic acid cycle − 28 13
tricarboxylic acid cycle intermediate metabolism − 18 9
glycine metabolism − 10 7
coenzyme metabolism − 153 32
amino acid biosynthesis − 136 6
catabolism − 404 55
biosynthesis − 966 37
coenzyme catabolism − 30 13
amine biosynthesis − 144 6
energy derivation by oxidation of organic compounds − 225 37
carbohydrate catabolism − 71 12
hexose metabolism − 112 23
hexose catabolism − 48 11
carboxylic acid metabolism − 353 37
cellular catabolism − 382 52
cellular carbohydrate metabolism − 227 41
nitrogen compound biosynthesis − 144 6
cellular carbohydrate catabolism − 71 12
alcohol catabolism − 56 11
acetyl−CoA catabolism − 28 13
monosaccharide catabolism − 53 11
cofactor metabolism − 181 34
cofactor catabolism − 31 13
tricarboxylic acid cycle − 28 13
amino acid biosynthesis − 96 6
mitochondrion − 986 22
cytosol − 313 13
cytosolic ribosome (sensu Eukaryota) − 130 10
cytosolic large ribosomal subunit (sensu Eukaryota) − 69 10
large ribosomal subunit − 111 10
mitochondrial part − 377 13
cytosolic part − 159 11
intracellular − 284 13
mitochondrion − 937 21
ribosome − 184 11
cytosolic large ribosomal subunit (sensu Eukaryota) − 69 10
ribonucleoprotein complex − 236 11
structural constituent of ribosome − 203 11
iron ion transporter activity − 10 3
structural constituent of ribosome − 203 11
C−compound and carbohydrate metabolism − 500 71 70
C−compound and carbohydrate utilization − 380 54
C−compound, carbohydrate catabolism − 115 25
sugar, glucoside, polyol and carboxylate catabolism − 80 19
ENERGY − 348 52
glycolysis and gluconeogenesis − 59 11
tricarboxylic−acid pathway (citrate cycle, Krebs cycle, TCA cycle) − 28 13
mitochondrion − 169 11
UNCLASSIFIED PROTEINS − 1454 31 37
Citrate cycle (TCA cycle) − 29 13
Glyoxylate and dicarboxylate metabolism − 14 8
Ribosome − 134 11
SIP4 − 11 8
GAL4 − 20 10 9
SKN7 − 171 11
GO_BP_comp
GO_BP_leaf
GO_CC_comp
GO_CC_leaf
GO_MF_comp
GO_MF_leaf
MIPS
KEGG
TF
452 356 102
69 39 30
220 160 60
346 190 156
28 14 14
73 45 28
13 5 8
77 36 41
73 3 70
C−source
C−source − Acetate
C−source − Galactose
C−source − Acetate / Ethanol
C−source − Acetate / Galactose
C−source − Glucose / Maltose
C−source − Glucose & N−source − Ammonium sulfate
C−source − Glucose & Limiting element − Carbon
C−source − Glucose & pH − 5

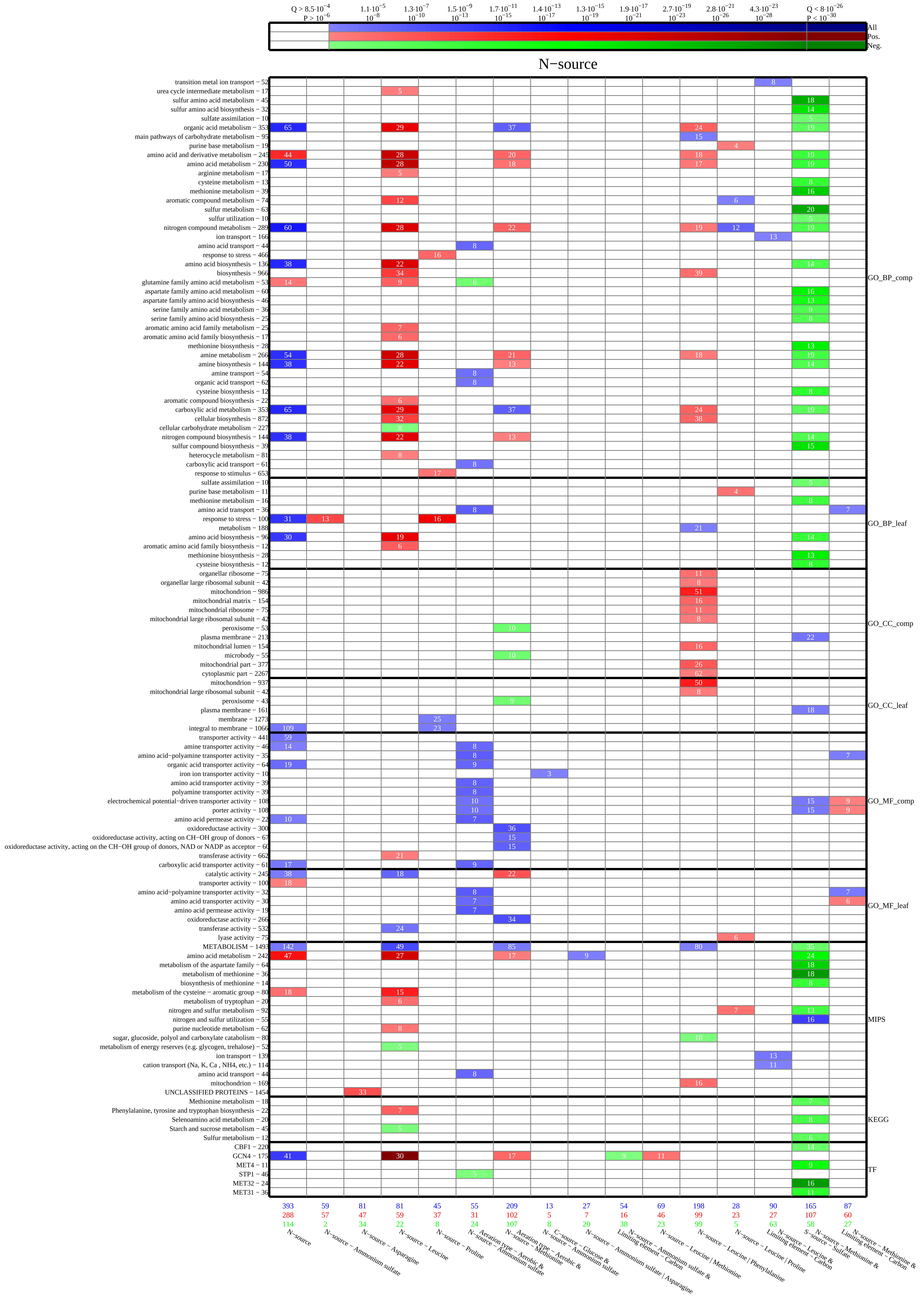

N−source
Q > 8.5·10−4 P > 10−6
Q < 8·10−26 P < 10−30
All
Pos.
Neg.
GO_BP_comp
GO_BP_leaf
GO_CC_comp
GO_CC_leaf
GO_MF_comp
GO_MF_leaf
MIPS
KEGG
TF
N−source
N−source − Ammonium sulfate
N−source − Asparagine
N−source − Leucine
N−source − Proline
Aeration type − Aerobic & N−source − Ammonium sulfate
Aeration type − Aerobic & N−source − Methionine
C−source − Glucose & N−source − Ammonium sulfate
N−source − Ammonium sulfate | Asparagine
N−source − Ammonium sulfate & Limiting element − Carbon
N−source − Leucine | Methionine
N−source − Leucine | Phenylalanine
N−source − Leucine | Proline
N−source − Leucine & Limiting element − Carbon
N−source − Methionine & S−source − Sulfate
N−source − Methionine & Limiting element − Carbon

	Q > 8.5·10^−4 P > 10^−6	1.1·10^−5 10^−8	1.3·10^−7 10^−10	1.5·10^−9 10^−13	1.7·10^−11 10^−15	1.4·10^−13 10^−17	1.3·10^−15 10^−19	1.9·10^−17 10^−21	2.7·10^−19 10^−23	2.8·10^−21 10^−26	4.3·10^−23 10^−28	Q < 8·10^−26 P < 10^−30
All												
Pos.												
Neg.												

S−source

Term	S−source − Methionine	S−source − Methionine & Limiting element − Carbon	S−source − Methionine & Limiting element − Nitrogen	N−source − Methionine & S−source − Sulfate	S−source − Sulfate & Limiting element − Nitrogen	S−source − Sulfate & Protocol − A	Category
sulfur amino acid metabolism − 45		16	9	18			GO_BP_comp
sulfur amino acid biosynthesis − 32		12	8	14			GO_BP_comp
sulfate assimilation − 10		5		5			GO_BP_comp
organic acid metabolism − 353		20		19			GO_BP_comp
protein biosynthesis − 462	49						GO_BP_comp
amino acid and derivative metabolism − 245		18	18	19			GO_BP_comp
amino acid metabolism − 230		18	18	19			GO_BP_comp
cysteine metabolism − 13		7		8			GO_BP_comp
methionine metabolism − 39		15	7	16			GO_BP_comp
sulfur metabolism − 63		20	12	20			GO_BP_comp
sulfur utilization − 10		5		5			GO_BP_comp
nitrogen compound metabolism − 289		20	21	19	13		GO_BP_comp
amino acid transport − 44		8			8		GO_BP_comp
amino acid biosynthesis − 136		16	10	14			GO_BP_comp
aspartate family amino acid metabolism − 60		15		16			GO_BP_comp
aspartate family amino acid biosynthesis − 46		11	7	13			GO_BP_comp
serine family amino acid metabolism − 36		8		9			GO_BP_comp
serine family amino acid biosynthesis − 25		7		8			GO_BP_comp
methionine biosynthesis − 28		11	6	13			GO_BP_comp
amine metabolism − 266		18	20	19			GO_BP_comp
amine biosynthesis − 144		16	10	14			GO_BP_comp
amine transport − 54		9			8		GO_BP_comp
organic acid transport − 62		9			9		GO_BP_comp
cysteine biosynthesis − 12		7		8			GO_BP_comp
carboxylic acid metabolism − 353		20		19			GO_BP_comp
nitrogen compound biosynthesis − 144		16	10	14			GO_BP_comp
sulfur compound biosynthesis − 39		14	8	15			GO_BP_comp
carboxylic acid transport − 61		9			9		GO_BP_comp
sulfate assimilation − 10		5		5			GO_BP_leaf
methionine metabolism − 16		9		8			GO_BP_leaf
amino acid transport − 36		7			7		GO_BP_leaf
amino acid biosynthesis − 96		12	9	14			GO_BP_leaf
methionine biosynthesis − 28		11	6	13			GO_BP_leaf
cysteine biosynthesis − 12		7		8			GO_BP_leaf
cytosolic ribosome (sensu Eukaryota) − 130	25						GO_CC_comp
plasma membrane − 213				22			GO_CC_comp
organelle part − 1965	134						GO_CC_comp
cytosolic part − 159	25						GO_CC_comp
intracellular organelle part − 1965	134						GO_CC_comp
plasma membrane − 161				18			GO_CC_leaf
ribonucleoprotein complex − 236	31						GO_CC_leaf
amine transporter activity − 46		8			8		GO_MF_comp
amino acid−polyamine transporter activity − 35		7			8		GO_MF_comp
organic acid transporter activity − 64					9		GO_MF_comp
amino acid transporter activity − 39					8		GO_MF_comp
polyamine transporter activity − 39		8			8		GO_MF_comp
electrochemical potential−driven transporter activity − 108				15	10		GO_MF_comp
porter activity − 108				15	10		GO_MF_comp
amino acid permease activity − 22		6			7		GO_MF_comp
carboxylic acid transporter activity − 61					9		GO_MF_comp
amino acid−polyamine transporter activity − 32		7			7		GO_MF_leaf
amino acid transporter activity − 30		7			7		GO_MF_leaf
amino acid permease activity − 19		6			6		GO_MF_leaf
METABOLISM − 1493		34		35			MIPS
amino acid metabolism − 242		23	23	24			MIPS
metabolism of the aspartate family − 64		15	11	18			MIPS
metabolism of methionine − 36		15	7	18			MIPS
biosynthesis of methionine − 14		5	5	8			MIPS
metabolism of the cysteine − aromatic group − 80			11				MIPS
nitrogen and sulfur metabolism − 92		14	14	13	8		MIPS
nitrogen and sulfur utilization − 55		9	10	16			MIPS
PROTEIN SYNTHESIS − 441	47						MIPS
ribosome biogenesis − 286	35						MIPS
ribosomal proteins − 229	30						MIPS
amino acid transport − 44		8			9		MIPS
UNCLASSIFIED PROTEINS − 1454						88	MIPS
Methionine metabolism − 18		6		7			KEGG
Selenoamino acid metabolism − 20		7		8			KEGG
Sulfur metabolism − 12		6		6			KEGG
Ribosome − 134	22						KEGG
CBF1 − 220		13	13	14			TF
GLN3 − 103					13		TF
MET4 − 11		7		9			TF
MET32 − 24		13		16			TF
MET31 − 36		7		11			TF
(All)	303	93	106	165	76	150	
(Pos.)	290	33	57	107	52	144	
(Neg.)	13	60	49	58	24	6	

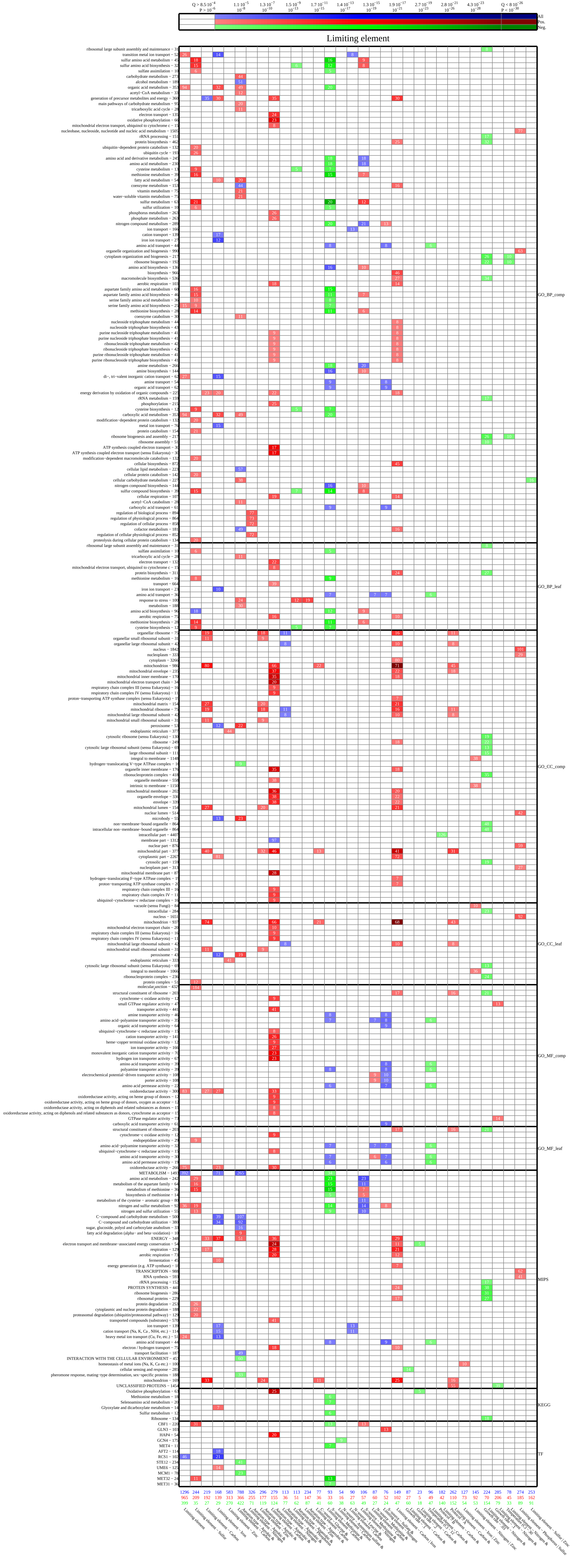
Limiting element
All
Pos.
Neg.
GO_BP_comp
GO_BP_leaf
GO_CC_comp
GO_CC_leaf
GO_MF_comp
GO_MF_leaf
MIPS
KEGG
TF

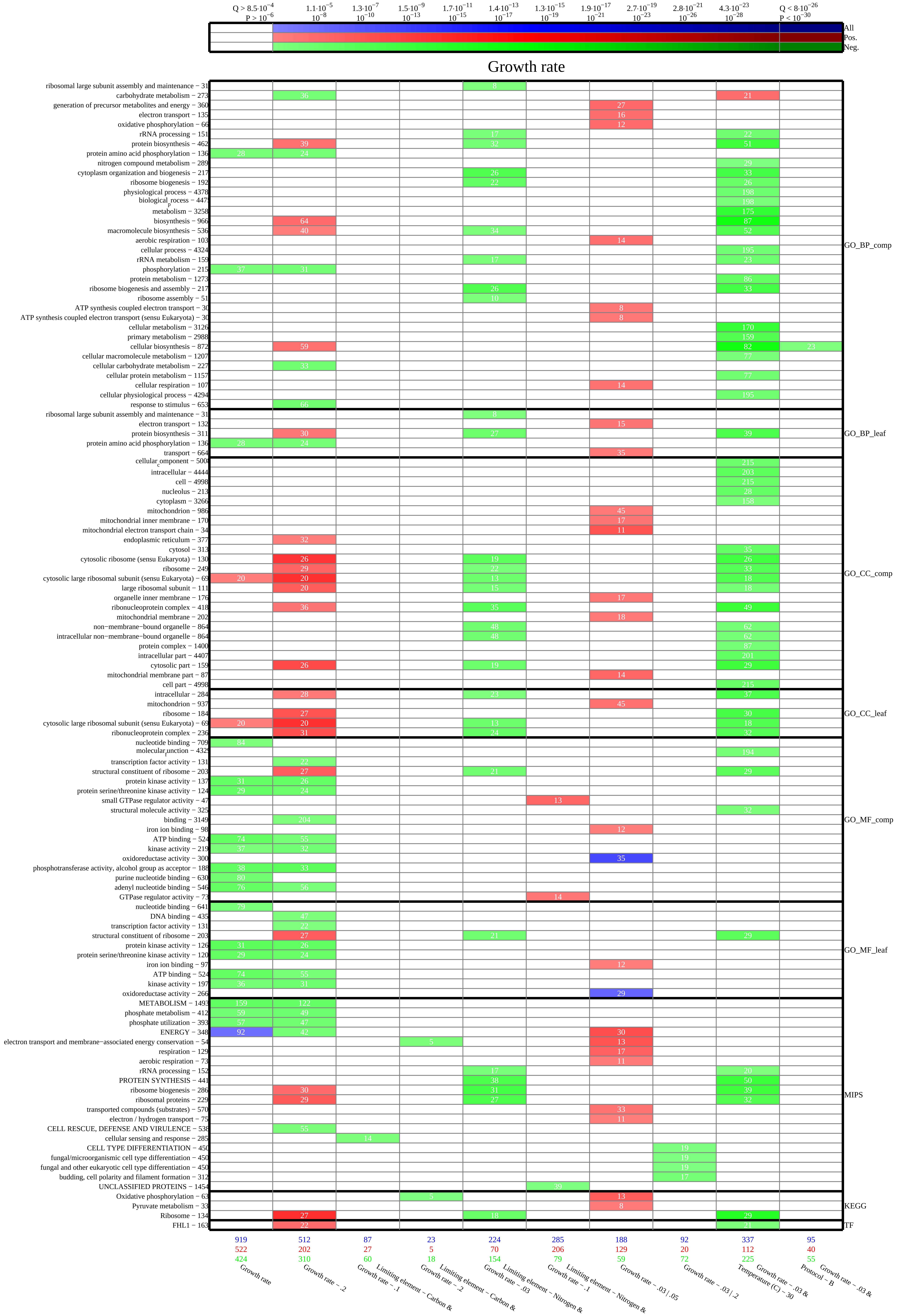
Q > 8.5·10^−4 P > 10^−6
1.1·10^−5 10^−8
1.3·10^−7 10^−10
1.5·10^−9 10^−13
1.7·10^−11 10^−15
1.4·10^−13 10^−17
1.3·10^−15 10^−19
1.9·10^−17 10^−21
2.7·10^−19 10^−23
2.8·10^−21 10^−26
4.3·10^−23 10^−28
Q < 8·10^−26 P < 10^−30
All
Pos.
Neg.
Growth rate
ribosomal large subunit assembly and maintenance − 31 8
carbohydrate metabolism − 273 36 21
generation of precursor metabolites and energy − 360 27
electron transport − 135 16
oxidative phosphorylation − 66 12
rRNA processing − 151 17 22
protein biosynthesis − 462 39 32 51
protein amino acid phosphorylation − 136 28 24
nitrogen compound metabolism − 289 29
cytoplasm organization and biogenesis − 217 26 33
ribosome biogenesis − 192 22 26
physiological process − 4378 198
biological_process − 4475 198
metabolism − 3258 175
biosynthesis − 966 64 87
macromolecule biosynthesis − 536 40 34 52
aerobic respiration − 103 14
cellular process − 4324 195
rRNA metabolism − 159 17 23
phosphorylation − 215 37 31
protein metabolism − 1273 86
ribosome biogenesis and assembly − 217 26 33
ribosome assembly − 51 10
ATP synthesis coupled electron transport − 30 8
ATP synthesis coupled electron transport (sensu Eukaryota) − 30 8
cellular metabolism − 3126 170
primary metabolism − 2988 159
cellular biosynthesis − 872 59 82 23
cellular macromolecule metabolism − 1207 77
cellular carbohydrate metabolism − 227 33
cellular protein metabolism − 1157 77
cellular respiration − 107 14
cellular physiological process − 4294 195
response to stimulus − 653 66
GO_BP_comp
ribosomal large subunit assembly and maintenance − 31 8
electron transport − 132 15
protein biosynthesis − 311 30 27 39
protein amino acid phosphorylation − 136 28 24
transport − 664 35
GO_BP_leaf
cellular_component − 5008 215
intracellular − 4444 203
cell − 4998 215
nucleolus − 213 28
cytoplasm − 3266 158
mitochondrion − 986 45
mitochondrial inner membrane − 170 17
mitochondrial electron transport chain − 34 11
endoplasmic reticulum − 377 32
cytosol − 313 35
cytosolic ribosome (sensu Eukaryota) − 130 26 19 26
ribosome − 249 29 22 33
cytosolic large ribosomal subunit (sensu Eukaryota) − 69 20 20 13 18
large ribosomal subunit − 111 20 15 18
organelle inner membrane − 176 17
ribonucleoprotein complex − 418 36 35 49
mitochondrial membrane − 202 18
non−membrane−bound organelle − 864 48 62
intracellular non−membrane−bound organelle − 864 48 62
protein complex − 1400 87
intracellular part − 4407 201
cytosolic part − 159 26 19 29
mitochondrial membrane part − 87 14
cell part − 4998 215
GO_CC_comp
intracellular − 284 28 23 37
mitochondrion − 937 45
ribosome − 184 27 30
cytosolic large ribosomal subunit (sensu Eukaryota) − 69 20 20 13 18
ribonucleoprotein complex − 236 31 24 32
GO_CC_leaf
nucleotide binding − 709 84
molecular_function − 4329 194
transcription factor activity − 131 22
structural constituent of ribosome − 203 27 21 29
protein kinase activity − 137 31 26
protein serine/threonine kinase activity − 124 29 24
small GTPase regulator activity − 47 13
structural molecule activity − 325 32
binding − 3149 204
iron ion binding − 98 12
ATP binding − 524 74 55
kinase activity − 219 37 32
oxidoreductase activity − 300 35
phosphotransferase activity, alcohol group as acceptor − 188 38 33
purine nucleotide binding − 630 80
adenyl nucleotide binding − 546 76 56
GTPase regulator activity − 73 14
GO_MF_comp
nucleotide binding − 641 79
DNA binding − 435 47
transcription factor activity − 131 22
structural constituent of ribosome − 203 27 21 29
protein kinase activity − 126 31 26
protein serine/threonine kinase activity − 120 29 24
iron ion binding − 97 12
ATP binding − 524 74 55
kinase activity − 197 36 31
oxidoreductase activity − 266 29
GO_MF_leaf
METABOLISM − 1493 159 122
phosphate metabolism − 412 59 49
phosphate utilization − 393 57 47
ENERGY − 348 92 42 30
electron transport and membrane−associated energy conservation − 54 5 13
respiration − 129 17
aerobic respiration − 73 11
rRNA processing − 152 17 20
PROTEIN SYNTHESIS − 441 38 50
ribosome biogenesis − 286 30 31 39
ribosomal proteins − 229 29 27 32
transported compounds (substrates) − 570 33
electron / hydrogen transport − 75 11
CELL RESCUE, DEFENSE AND VIRULENCE − 538 55
cellular sensing and response − 285 14
CELL TYPE DIFFERENTIATION − 450 19
fungal/microorganismic cell type differentiation − 450 19
fungal and other eukaryotic cell type differentiation − 450 19
budding, cell polarity and filament formation − 312 17
UNCLASSIFIED PROTEINS − 1454 39
MIPS
Oxidative phosphorylation − 63 5 13
Pyruvate metabolism − 33 8
Ribosome − 134 27 18 29
KEGG
FHL1 − 163 22 21
TF
919 522 424
512 202 310
87 27 60
23 5 18
224 70 154
285 206 79
188 129 59
92 20 72
337 112 225
95 40 55
Growth rate
Growth rate − .2
Growth rate − .1 Limiting element − Carbon &
Growth rate − .2 Limiting element − Carbon &
Growth rate − .03 Limiting element − Carbon &
Growth rate − .1 Limiting element − Nitrogen &
Growth rate − .03 / .05 Limiting element − Nitrogen &
Growth rate − .03 / .05
Growth rate − .03 / .2
Temperature (C) − 30 Growth rate − .03 &
Protocol − B Growth rate − .03 &

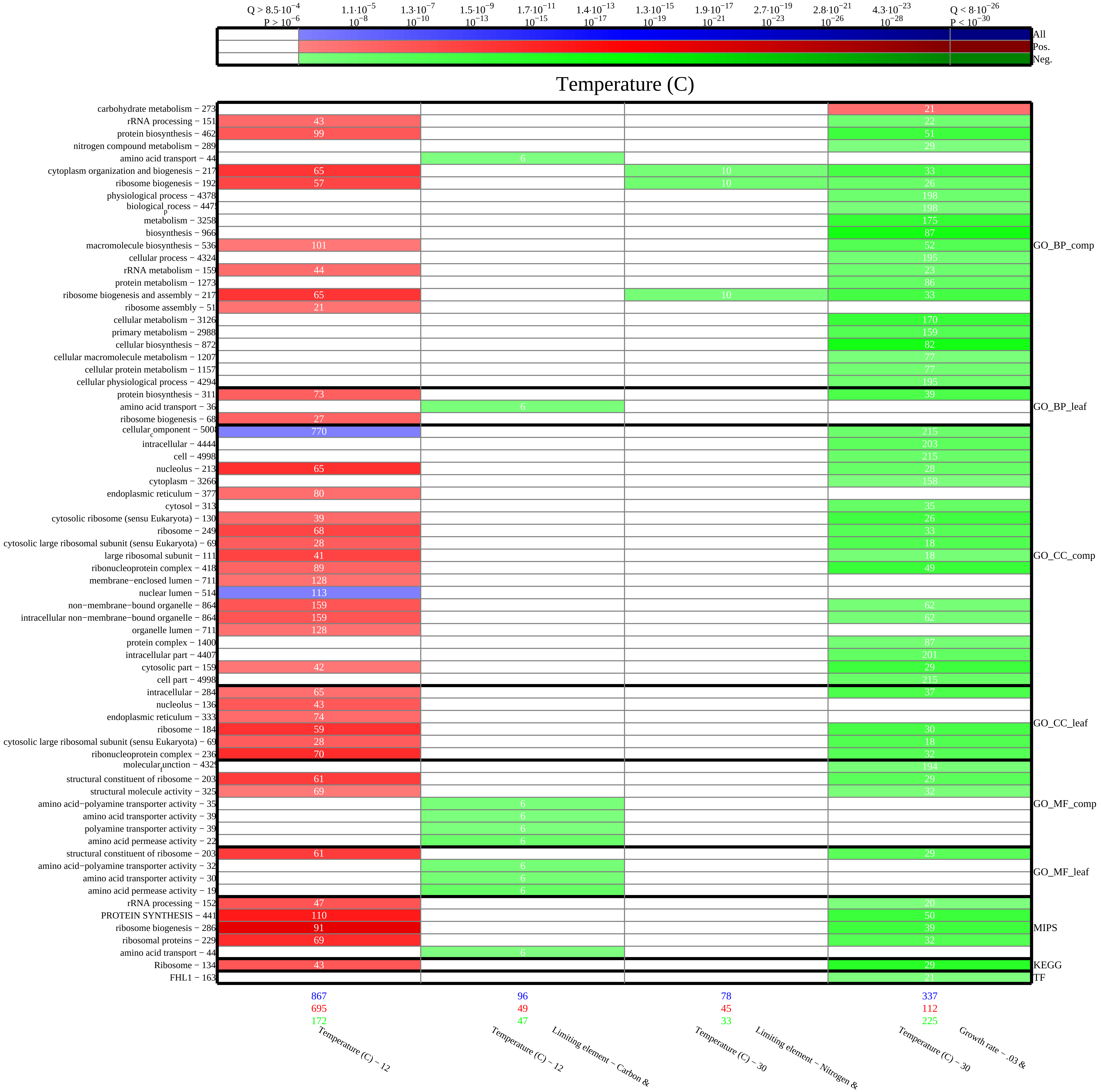

Q > 8.5·10^−4 P > 10^−6
1.1·10^−5 10^−8
1.3·10^−7 10^−10
1.5·10^−9 10^−13
1.7·10^−11 10^−15
1.4·10^−13 10^−17
1.3·10^−15 10^−19
1.9·10^−17 10^−21
2.7·10^−19 10^−23
2.8·10^−21 10^−26
4.3·10^−23 10^−28
Q < 8·10^−26 P < 10^−30
All
Pos.
Neg.
Temperature (C)
carbohydrate metabolism − 273 | 21
rRNA processing − 151 | 43 | 22
protein biosynthesis − 462 | 99 | 51
nitrogen compound metabolism − 289 | 29
amino acid transport − 44 | 6
cytoplasm organization and biogenesis − 217 | 65 | 10 | 33
ribosome biogenesis − 192 | 57 | 10 | 26
physiological process − 4378 | 198
biological process − 4475 | 198
metabolism − 3258 | 175
biosynthesis − 966 | 87
macromolecule biosynthesis − 536 | 101 | 52
cellular process − 4324 | 195
rRNA metabolism − 159 | 44 | 23
protein metabolism − 1273 | 86
ribosome biogenesis and assembly − 217 | 65 | 10 | 33
ribosome assembly − 51 | 21
cellular metabolism − 3126 | 170
primary metabolism − 2988 | 159
cellular biosynthesis − 872 | 82
cellular macromolecule metabolism − 1207 | 77
cellular protein metabolism − 1157 | 77
cellular physiological process − 4294 | 195
GO_BP_comp
protein biosynthesis − 311 | 73 | 39
amino acid transport − 36 | 6
ribosome biogenesis − 68 | 27
GO_BP_leaf
cellular component − 5008 | 770 | 215
intracellular − 4444 | 203
cell − 4998 | 215
nucleolus − 213 | 65 | 28
cytoplasm − 3266 | 158
endoplasmic reticulum − 377 | 80
cytosol − 313 | 35
cytosolic ribosome (sensu Eukaryota) − 130 | 39 | 26
ribosome − 249 | 68 | 33
cytosolic large ribosomal subunit (sensu Eukaryota) − 69 | 28 | 18
large ribosomal subunit − 111 | 41 | 18
ribonucleoprotein complex − 418 | 89 | 49
membrane−enclosed lumen − 711 | 128
nuclear lumen − 514 | 113
non−membrane−bound organelle − 864 | 159 | 62
intracellular non−membrane−bound organelle − 864 | 159 | 62
organelle lumen − 711 | 128
protein complex − 1400 | 87
intracellular part − 4407 | 201
cytosolic part − 159 | 42 | 29
cell part − 4998 | 215
GO_CC_comp
intracellular − 284 | 65 | 37
nucleolus − 136 | 43
endoplasmic reticulum − 333 | 74
ribosome − 184 | 59 | 30
cytosolic large ribosomal subunit (sensu Eukaryota) − 69 | 28 | 18
ribonucleoprotein complex − 236 | 70 | 32
GO_CC_leaf
molecular function − 4329 | 194
structural constituent of ribosome − 203 | 61 | 29
structural molecule activity − 325 | 69 | 32
amino acid−polyamine transporter activity − 35 | 6
amino acid transporter activity − 39 | 6
polyamine transporter activity − 39 | 6
amino acid permease activity − 22 | 6
GO_MF_comp
structural constituent of ribosome − 203 | 61 | 29
amino acid−polyamine transporter activity − 32 | 6
amino acid transporter activity − 30 | 6
amino acid permease activity − 19 | 6
GO_MF_leaf
rRNA processing − 152 | 47 | 20
PROTEIN SYNTHESIS − 441 | 110 | 50
ribosome biogenesis − 286 | 91 | 39
ribosomal proteins − 229 | 69 | 32
amino acid transport − 44 | 6
MIPS
Ribosome − 134 | 43 | 29
KEGG
FHL1 − 163 | 21
TF
867 695 172
96 49 47
78 45 33
337 112 225
Temperature (C) − 12
Temperature (C) − 12 Limiting element − Carbon &
Temperature (C) − 30 Limiting element − Nitrogen &
Temperature (C) − 30 Growth rate − .03 &

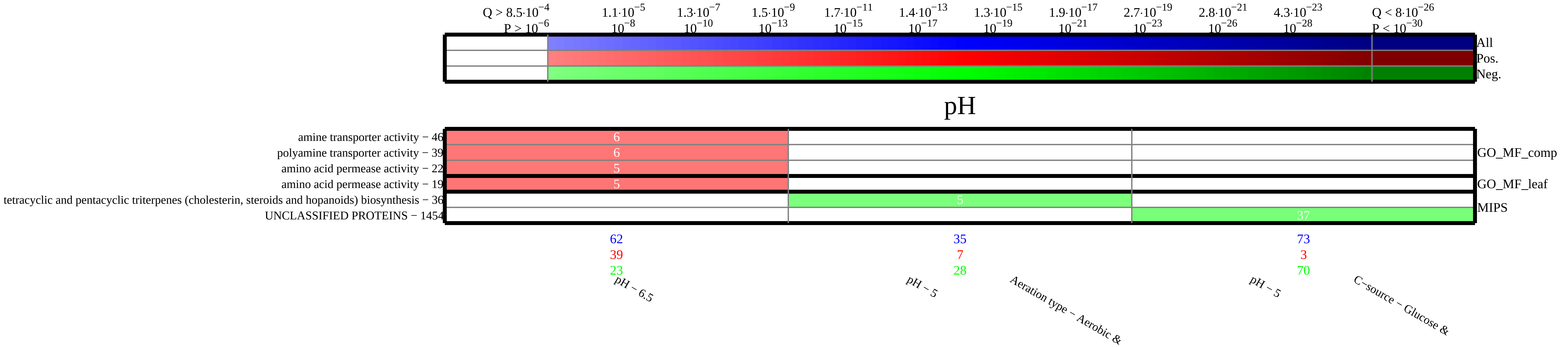
Q > 8.5·10−4
P > 10−6
1.1·10−5
10−8
1.3·10−7
10−10
1.5·10−9
10−13
1.7·10−11
10−15
1.4·10−13
10−17
1.3·10−15
10−19
1.9·10−17
10−21
2.7·10−19
10−23
2.8·10−21
10−26
4.3·10−23
10−28
Q < 8·10−26
P < 10−30
All
Pos.
Neg.
pH
amine transporter activity − 46
polyamine transporter activity − 39
amino acid permease activity − 22
amino acid permease activity − 19
tetracyclic and pentacyclic triterpenes (cholesterin, steroids and hopanoids) biosynthesis − 36
UNCLASSIFIED PROTEINS − 1454
GO_MF_comp
GO_MF_leaf
MIPS
6
6
5
5
5
37
62
39
23
35
7
28
73
3
70
pH − 6.5
pH − 5
Aeration type − Aerobic &
pH − 5
C−source − Glucose &

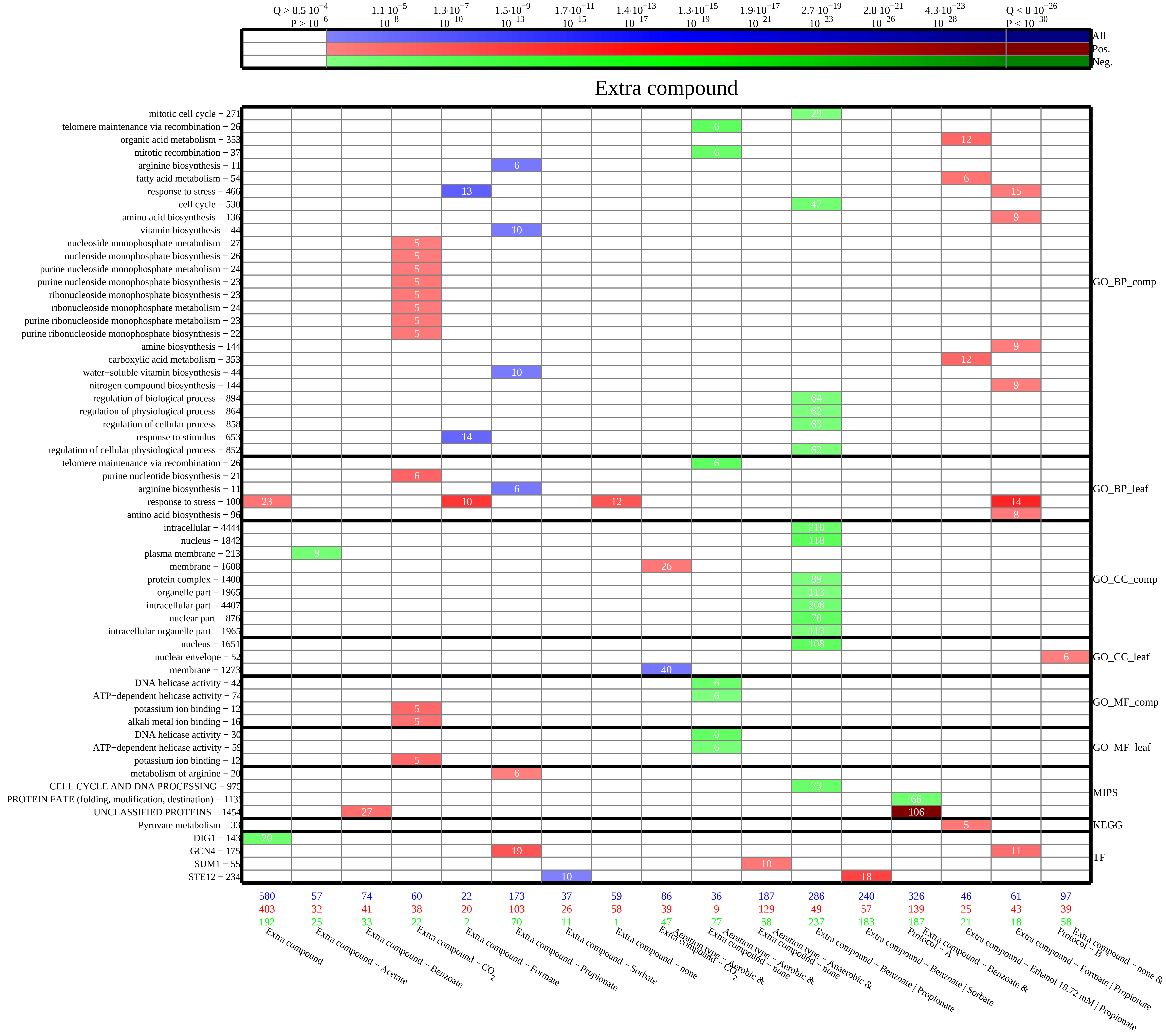
Q > 8.5·10^−4 P > 10^−6
1.1·10^−5 10^−8
1.3·10^−7 10^−10
1.5·10^−9 10^−13
1.7·10^−11 10^−15
1.4·10^−13 10^−17
1.3·10^−15 10^−19
1.9·10^−17 10^−21
2.7·10^−19 10^−23
2.8·10^−21 10^−26
4.3·10^−23 10^−28
Q < 8·10^−26 P < 10^−30
All
Pos.
Neg.
Extra compound
mitotic cell cycle − 271
telomere maintenance via recombination − 26
organic acid metabolism − 353
mitotic recombination − 37
arginine biosynthesis − 11
fatty acid metabolism − 54
response to stress − 466
cell cycle − 530
amino acid biosynthesis − 136
vitamin biosynthesis − 44
nucleoside monophosphate metabolism − 27
nucleoside monophosphate biosynthesis − 26
purine nucleoside monophosphate metabolism − 24
purine nucleoside monophosphate biosynthesis − 23
ribonucleoside monophosphate biosynthesis − 23
ribonucleoside monophosphate metabolism − 24
purine ribonucleoside monophosphate metabolism − 23
purine ribonucleoside monophosphate biosynthesis − 22
amine biosynthesis − 144
carboxylic acid metabolism − 353
water−soluble vitamin biosynthesis − 44
nitrogen compound biosynthesis − 144
regulation of biological process − 894
regulation of physiological process − 864
regulation of cellular process − 858
response to stimulus − 653
regulation of cellular physiological process − 852
GO_BP_comp
telomere maintenance via recombination − 26
purine nucleotide biosynthesis − 21
arginine biosynthesis − 11
response to stress − 100
amino acid biosynthesis − 96
GO_BP_leaf
intracellular − 4444
nucleus − 1842
plasma membrane − 213
membrane − 1608
protein complex − 1400
organelle part − 1965
intracellular part − 4407
nuclear part − 876
intracellular organelle part − 1965
GO_CC_comp
nucleus − 1651
nuclear envelope − 52
membrane − 1273
GO_CC_leaf
DNA helicase activity − 42
ATP−dependent helicase activity − 74
potassium ion binding − 12
alkali metal ion binding − 16
GO_MF_comp
DNA helicase activity − 30
ATP−dependent helicase activity − 59
potassium ion binding − 12
GO_MF_leaf
metabolism of arginine − 20
CELL CYCLE AND DNA PROCESSING − 975
PROTEIN FATE (folding, modification, destination) − 1135
UNCLASSIFIED PROTEINS − 1454
MIPS
Pyruvate metabolism − 33
KEGG
DIG1 − 143
GCN4 − 175
SUM1 − 55
STE12 − 234
TF
580 57 74 60 22 173 37 59 86 36 187 286 240 326 46 61 97
403 32 41 38 20 103 26 58 39 9 129 49 57 139 25 43 39
192 25 33 22 2 70 11 1 47 27 58 237 183 187 21 18 58
Extra compound
Extra compound − Acetate
Extra compound − Benzoate
Extra compound − CO2
Extra compound − Formate
Extra compound − Propionate
Extra compound − Sorbate
Extra compound − none
Aeration type − Aerobic & Extra compound − CO2
Aeration type − Aerobic & Extra compound − none
Aeration type − Anaerobic & Extra compound − none
Extra compound − Benzoate | Propionate
Extra compound − Benzoate | Sorbate
Extra compound − Benzoate & Protocol − A
Extra compound − Ethanol 18.72 mM | Propionate
Extra compound − Formate | Propionate
Extra compound − none & Protocol − B

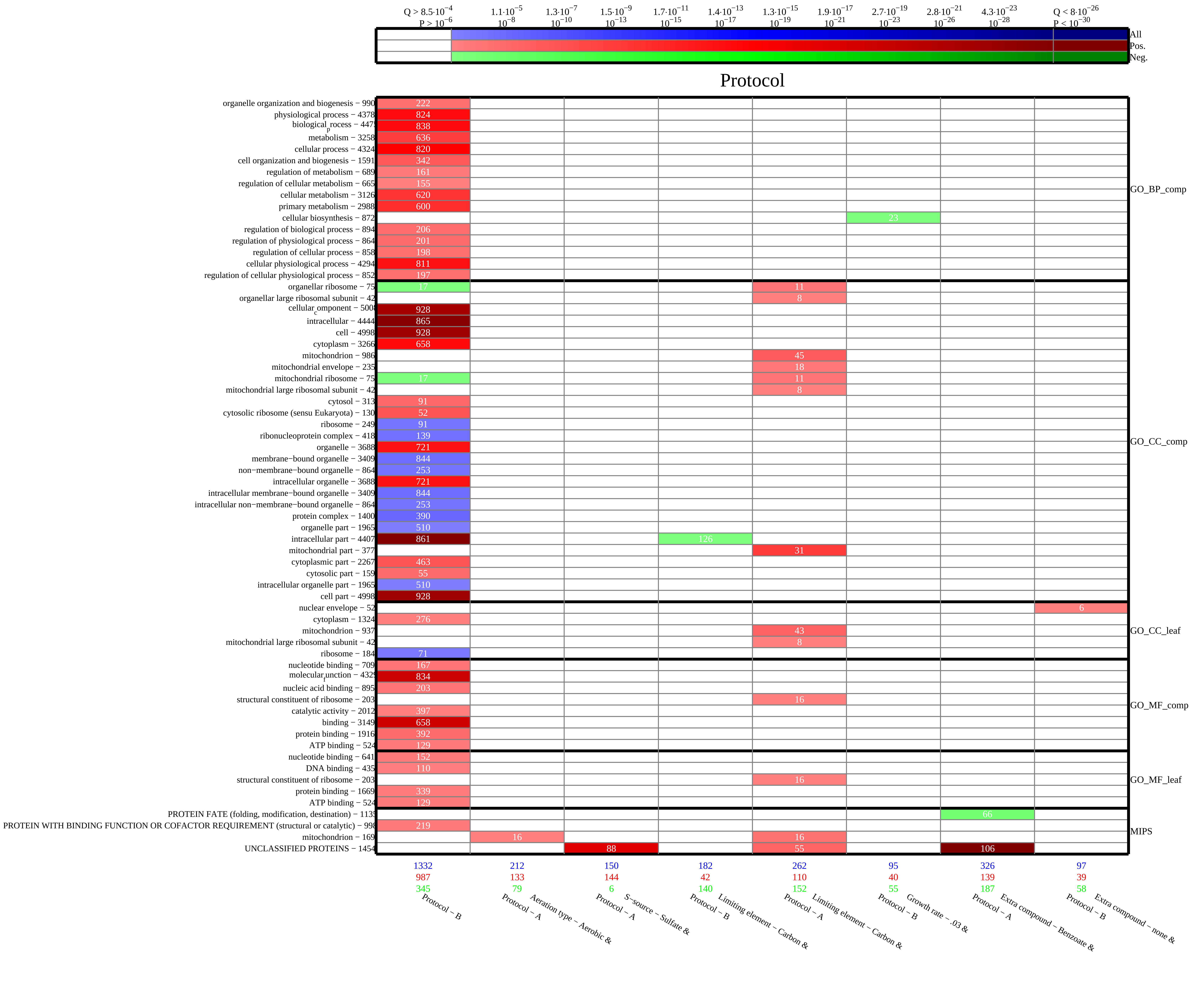

Q > 8.5·10^−4 P > 10^−6
1.1·10^−5 10^−8
1.3·10^−7 10^−10
1.5·10^−9 10^−13
1.7·10^−11 10^−15
1.4·10^−13 10^−17
1.3·10^−15 10^−19
1.9·10^−17 10^−21
2.7·10^−19 10^−23
2.8·10^−21 10^−26
4.3·10^−23 10^−28
Q < 8·10^−26 P < 10^−30
All
Pos.
Neg.
Protocol
organelle organization and biogenesis − 990: 222
physiological process − 4378: 824
biological process − 4475: 838
metabolism − 3258: 636
cellular process − 4324: 820
cell organization and biogenesis − 1591: 342
regulation of metabolism − 689: 161
regulation of cellular metabolism − 665: 155
cellular metabolism − 3126: 620
primary metabolism − 2988: 600
cellular biosynthesis − 872: 23
regulation of biological process − 894: 206
regulation of physiological process − 864: 201
regulation of cellular process − 858: 198
cellular physiological process − 4294: 811
regulation of cellular physiological process − 852: 197
GO_BP_comp
organellar ribosome − 75: 17, 11
organellar large ribosomal subunit − 42: 8
cellular component − 5008: 928
intracellular − 4444: 865
cell − 4998: 928
cytoplasm − 3266: 658
mitochondrion − 986: 45
mitochondrial envelope − 235: 18
mitochondrial ribosome − 75: 17, 11
mitochondrial large ribosomal subunit − 42: 8
cytosol − 313: 91
cytosolic ribosome (sensu Eukaryota) − 130: 52
ribosome − 249: 91
ribonucleoprotein complex − 418: 139
organelle − 3688: 721
membrane−bound organelle − 3409: 844
non−membrane−bound organelle − 864: 253
intracellular organelle − 3688: 721
intracellular membrane−bound organelle − 3409: 844
intracellular non−membrane−bound organelle − 864: 253
protein complex − 1400: 390
organelle part − 1965: 510
intracellular part − 4407: 861, 126
mitochondrial part − 377: 31
cytoplasmic part − 2267: 463
cytosolic part − 159: 55
intracellular organelle part − 1965: 510
cell part − 4998: 928
GO_CC_comp
nuclear envelope − 52: 6
cytoplasm − 1324: 276
mitochondrion − 937: 43
mitochondrial large ribosomal subunit − 42: 8
ribosome − 184: 71
GO_CC_leaf
nucleotide binding − 709: 167
molecular function − 4329: 834
nucleic acid binding − 895: 203
structural constituent of ribosome − 203: 16
catalytic activity − 2012: 397
binding − 3149: 658
protein binding − 1916: 392
ATP binding − 524: 129
GO_MF_comp
nucleotide binding − 641: 152
DNA binding − 435: 110
structural constituent of ribosome − 203: 16
protein binding − 1669: 339
ATP binding − 524: 129
GO_MF_leaf
PROTEIN FATE (folding, modification, destination) − 1135: 66
PROTEIN WITH BINDING FUNCTION OR COFACTOR REQUIREMENT (structural or catalytic) − 998: 219
mitochondrion − 169: 16, 16
UNCLASSIFIED PROTEINS − 1454: 88, 55, 106
MIPS
1332 987 345 Protocol − B
212 133 79 Protocol − A Aeration type − Aerobic &
150 144 6 Protocol − A S−source − Sulfate &
182 42 140 Protocol − B Limiting element − Carbon &
262 110 152 Protocol − A Limiting element − Carbon &
95 40 55 Protocol − B Growth rate − .03 &
326 139 187 Protocol − A Extra compound − Benzoate &
97 39 58 Protocol − B Extra compound − none &
