# Supplementary material for: Combinatorial effects of environmental parameters on transcriptional regulation in Saccharomyces cerevisiae: A quantitative analysis of a compendium of chemostat-based transcriptome data
Source: BMC Genomics. 2009 Jan 27;10:53. doi: 10.1186/1471-2164-10-53 (PMC2640415; doi:10.1186/1471-2164-10-53)
Supplement: Additional file 2 — Comparison between regression analysis including and excluding combinatorial effects. This file describes the comparison between the regression analysis including and excluding combinatorial effects by analyzing the explained variance and functional enrichment of clustered genes. [file 1471-2164-10-53-S2.pdf]

Comparison between regression analysis including and excluding combinatorial effects.

Explained variance

The regression analysis was repeated using only the single effects as predictors in the model. There are 38 single effects. The p-value cut-off to decide whether a predictor is significant is 0.05/38 (in stead of 0.05/227 when combinatorial effects are included). Figure I depicts the explained variance (including replicate variance) of all (6005) differentially expressed genes both for the regression model that employs single and combinatorial effects, indicated by R^{sc}, and the model that employs only the single effects, indicated by R^{s}. From this figure it is clearly visible that, in general, R^{sc} provides a better explanation of the variance in the expression pattern of a gene.

Enrichment analysis

R^{sc} and R^{s} were employed to reconstruct the expression pattern of the genes for which 70% of their variance could be explained by R^{sc} and/or R^{s}. (The reconstructed expression pattern of one gene is indicated by the shaded area in Figure 1 in the manuscript.) The genes (represented by their reconstructed expression patterns) were clustered and these clusters were consulted for overrepresentation of functional categories. In more detail: The genes were clustered using hierarchical clustering with correlation as the distance measure and average linkage as linking criterium. The resulting dendrogram was cut to arrive at 2, 5, 10, 25, 50 100, 150 and 200 clusters. Each of these 542 (= 2+5+…200) clusters was consulted for enrichment in each functional category in GO, MIPS, KEGG and TF binding data, which consists of more than ten genes, using the hypergeometric test. From each category we record the minimal p-value (maximum enrichment score) across all clusters. Besides performing this procedure on the reconstructed expression data (using either R^{sc} or R^{s}), also the raw gene expression data (averaged over the replicates), indicated by E, was used to compute enrichment values.

Figure II presents an overview of the results. From this it is evident that most functional categories are more enriched when clustered using R^{sc} when compared to R^{s}. Also, there is only a slight difference between E and R^{sc}, indicating that the regression model captures most of the (known) functionality of the genes.

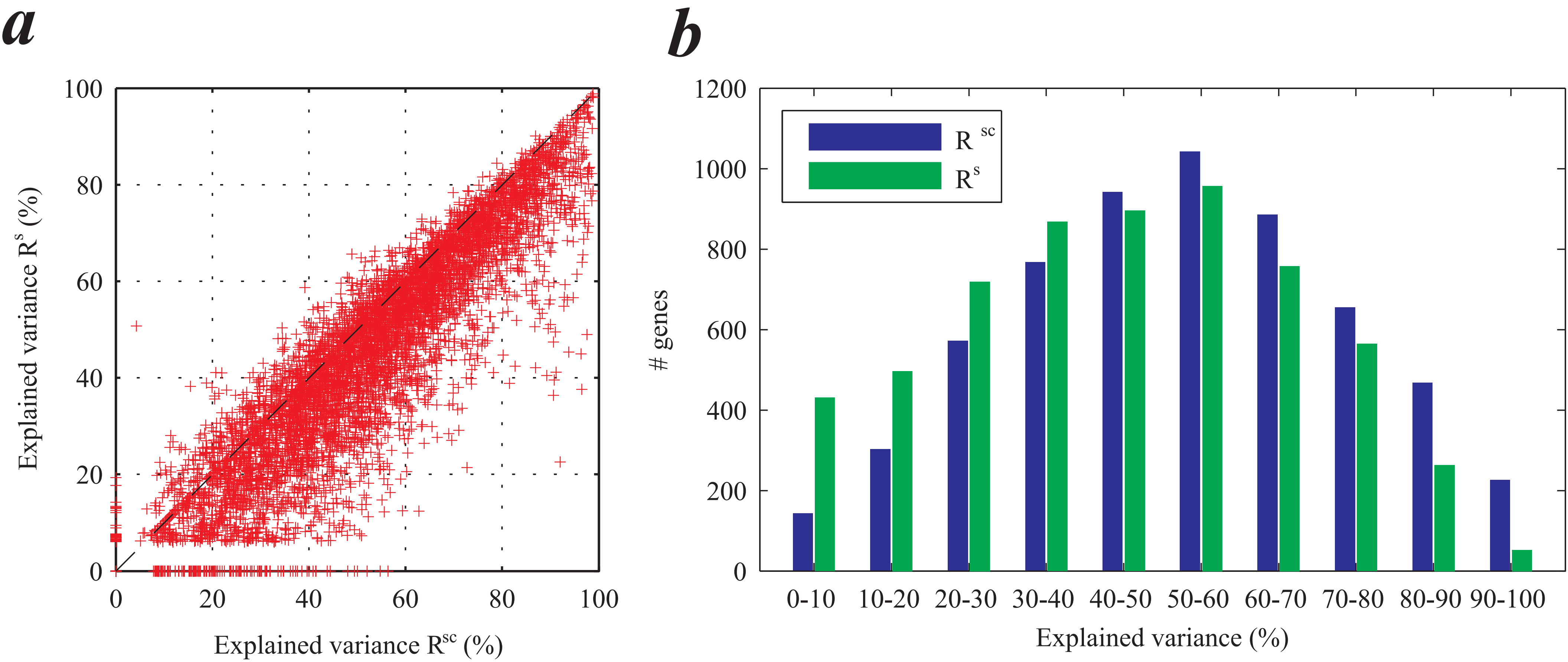

Figure I ***a*** Explained variance of all 6005 differentially expressed genes using regression including the combinatorial effects (R^{sc}) and excluding the combinatorial effects (R^{s}). ***b*** Histogram plot indicating the explained variance for both methods.

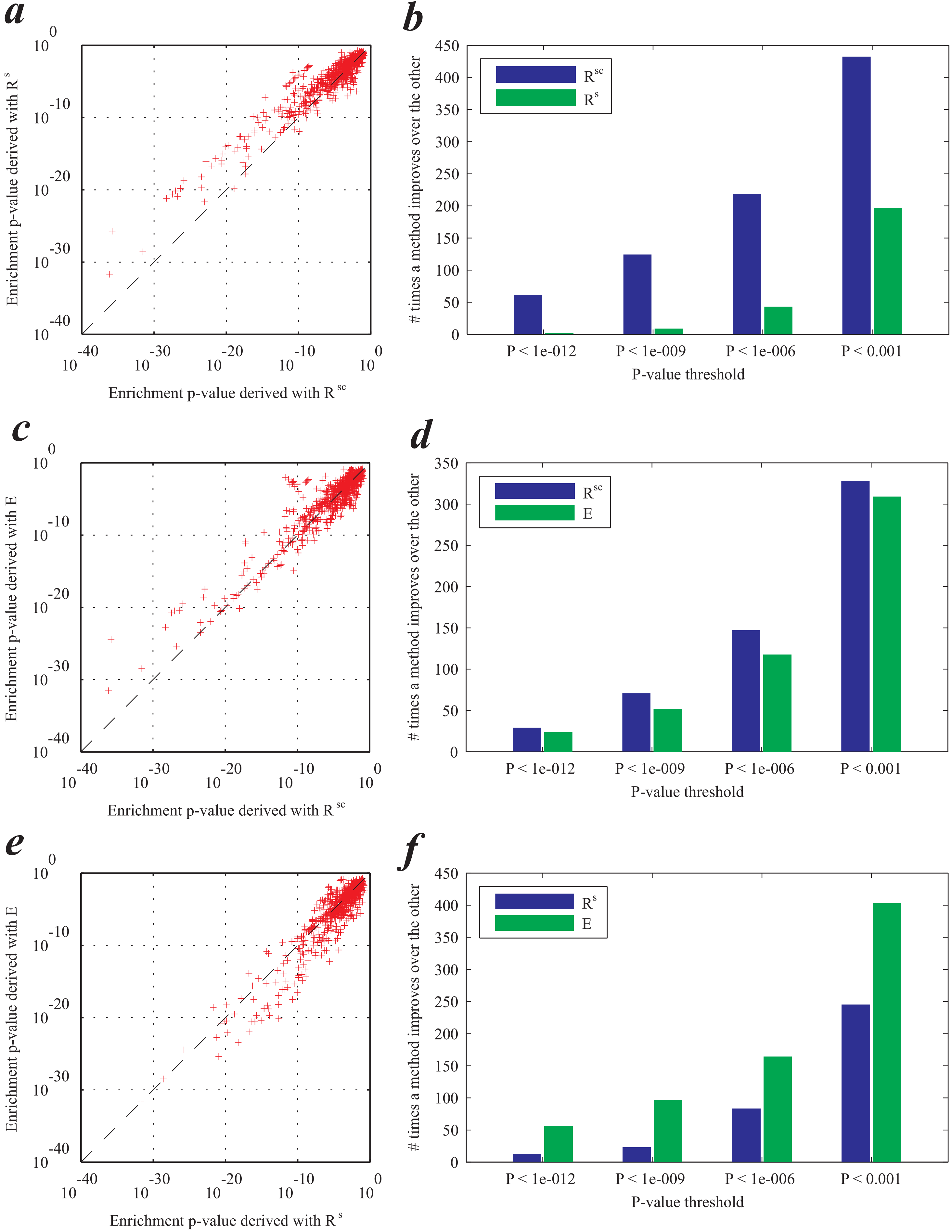

Figure II ***a*** Minimum p-value (maximum enrichment) of functional categories across all clusterings derived from the reconstructed expression pattern based on regression including the combinatorial effects (R^{sc}) and excluding the combinatorial effects (R^{s}). ***b*** Histogram plot indicating the number of times one method (R^{sc} or R^{s}) leads to better enrichment (lower p-value) of a functional category given that the enrichment of this functional category is below a p-value threshold (x-axis) for at least one of both methods. ***c, d*** Similar to ***a, b*** However, in stead of comparing R^{sc} and R^{s}, now R^{sc} is compared with E. For E, clustering is based on the raw expression levels. ***e, f*** Similar to ***a, b***, comparing E and R^{s}.
