# Supplementary material for: Combinatorial effects of environmental parameters on transcriptional regulation in Saccharomyces cerevisiae: A quantitative analysis of a compendium of chemostat-based transcriptome data
Source: BMC Genomics. 2009 Jan 27;10:53. doi: 10.1186/1471-2164-10-53 (PMC2640415; doi:10.1186/1471-2164-10-53)
Supplement: Additional file 3 — Details of the different protocols. This file states the details of the different protocols to prepare samples for hybridization to the microarray. References to the corresponding manuals are given. Also, advantages and disadvantages of both protocols are given. [file 1471-2164-10-53-S3.pdf]

Protocol A:

Harvest ~ 700 µg total RNA.
Perform the One-Cycle Target Labeling and Control Reagents Eukaryotic Target Preparation steps as described in [1]. This includes the Poly-A mRNA Isolation step. After purification, there is ~ 11 µg purified mRNA, of which 3-5 µg will be used in the following steps.

Disadvantages:
-time,
-money,
-since different mRNA's have different lengths of the poly A-tail, the purification step that filters on the mRNA poly-A tail could form a bias.
Advantages:
-rRNA and tRNA cannot interfere with cDNA synthesis.

Protocol B:

Harvest ~ 15 µg total RNA.
Perform the One-Cycle Target Labeling and Control Reagents Eukaryotic Target Preparation steps as described in [2]. However, do not perform the Poly-A mRNA Isolation step.

Disadvantages:
-rRNA and tRNA can interfere with cDNA synthesis.
Advantages:
-time,
-money,
-possible bias induced by filtering on the poly-A tails is circumvented.

[1] AffyMetrix, GeneChip Expression Analysis Technical Manual P/N 700219 rev 4, 2000
[2] AffyMetrix, GeneChip Expression Analysis Technical Manual P/N 701025 rev 5, 2000
