# Supplementary figures and images for: Combinatorial effects of environmental parameters on transcriptional regulation in Saccharomyces cerevisiae: A quantitative analysis of a compendium of chemostat-based transcriptome data
Source: BMC Genomics. 2009 Jan 27;10:53. doi: 10.1186/1471-2164-10-53 (PMC2640415; doi:10.1186/1471-2164-10-53)

Q =  $5.5 \cdot 10^{-3}$  P =  $10^{-5}$

Q =  $8 \cdot 10^{-26}$  P =  $10^{-30}$

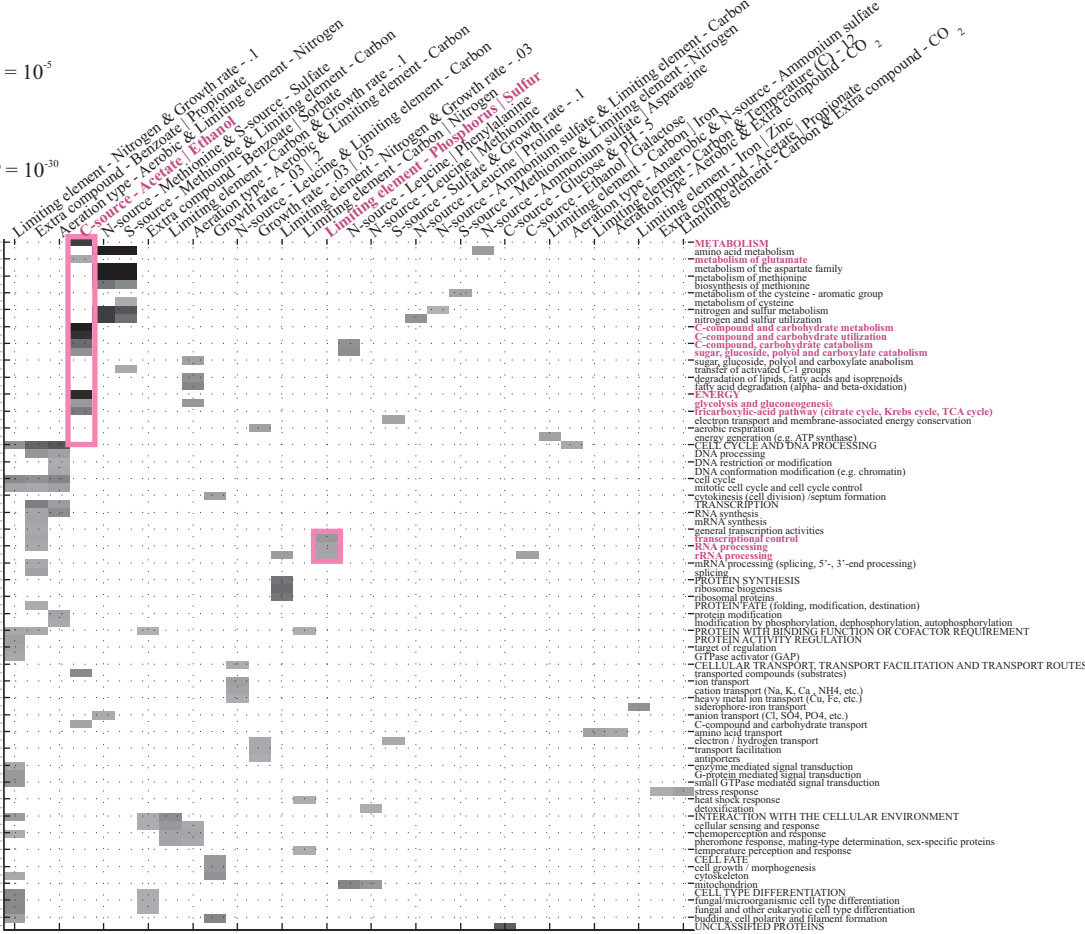

Supplement: Additional file 4 — MIPS functional categories that are specifically influenced by combinatorial effects. The significant category-effect-pairs are depicted by the dark boxes. The grey value of a box indicates the enrichment p-value and associated false discovery rate (q-value). On the right of the figure are the names of the significant MIPS categories; on the left is the hierarchy within these categories; the combinatorial effects are listed above the figure. The two cases that are discussed in the text are indicated by the magenta boxes. The corresponding MIPS categories and cultivation parameters are printed in bold and magenta. [file 1471-2164-10-53-S4.pdf]

## Array ID

## Description of the predictors

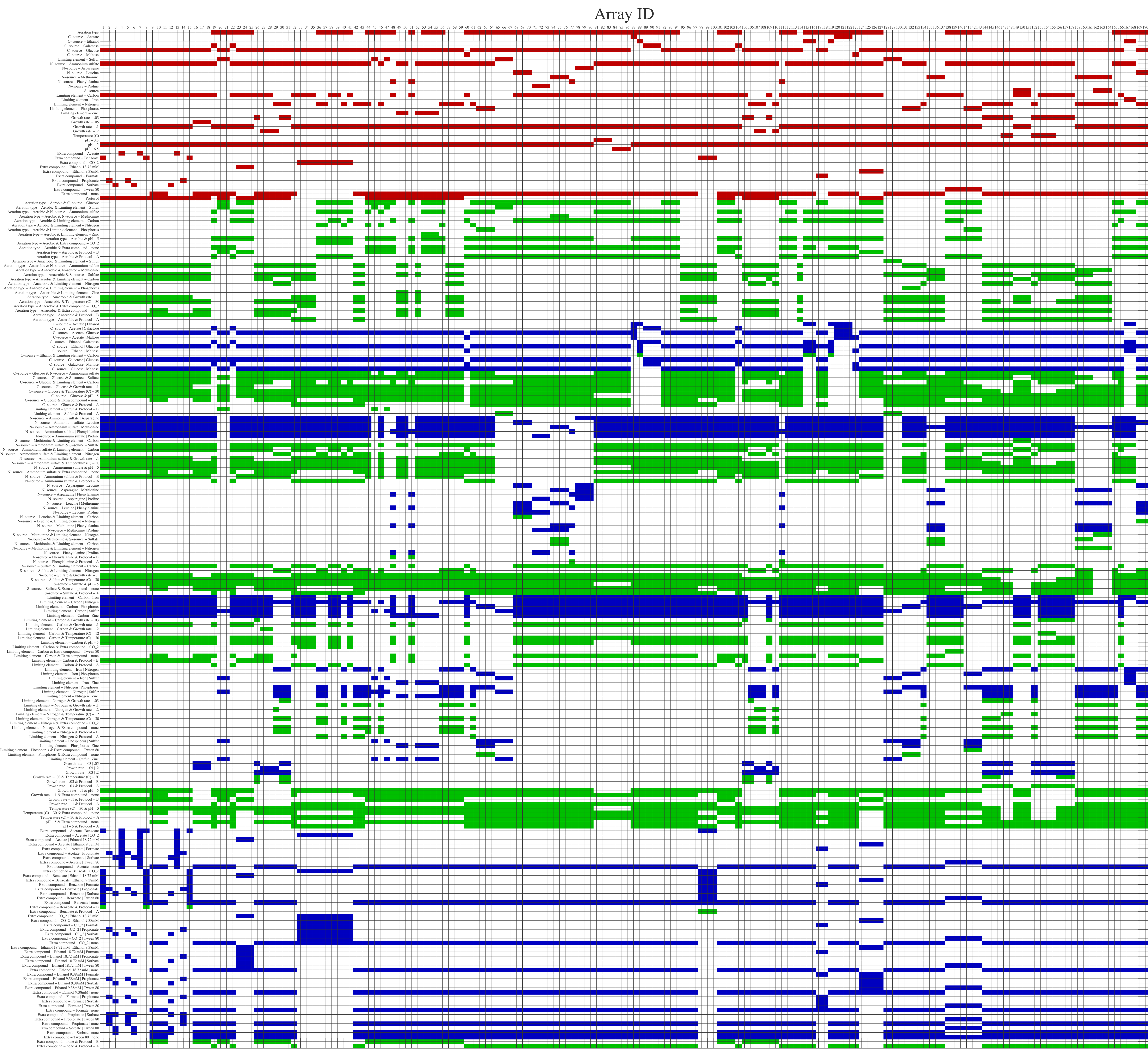

Supplement: Additional file 8 — Visualization of designmatrix D. This file is a visualization of the [170 × 227] binary designmatrix D. This matrix indicates for each of the 170 arrays/cultivations under which of the 227 (combinatorial) cultivation parameters the yeast was grown. These are marked by the non-white elements, which represent the 1's of D. (White elements represent the 0's.) For visibility, the transpose of the matrix is displayed. The x-axis contains the 170 array IDs; the y-axis displays the descriptions of the 227 predictors (which include 38 single effects (in red), 101 AND effects (in green) and 88 OR effects (in blue)). [file 1471-2164-10-53-S8.pdf]
