# Supplementary material for: Combinatorial effects of environmental parameters on transcriptional regulation in Saccharomyces cerevisiae: A quantitative analysis of a compendium of chemostat-based transcriptome data
Source: BMC Genomics. 2009 Jan 27;10:53. doi: 10.1186/1471-2164-10-53 (PMC2640415; doi:10.1186/1471-2164-10-53)

Of the 54 dubious genes, we selected the genes, for which aeration or an interaction effect between carbon limitation and aeration, were signicant predictors according to the regression model. These 35 genes were screened against all quantified peptides as identified in [1].

Three were found:

1. YAR073W

The IMD family consist of five ORF's:

Systematic name	Gene name	SGD Status	Position
YAR073W	IMD1	Dubious	ChrI:<br>227733 to 228944
YAR075W	---	Dubious	ChrI:<br>228835 to 229308
YHR216W	IMD2	Verified	ChrVIII:<br>554396 to 555967
YLR432W	IMD3	Verified	ChrXII:<br>1002554 to 1004125
YML056C	IMD4	Verified	ChrXIII:<br>164176 to 162194

All these ORF's exhibit a large degree of sequence similarity. The probes on the microarray are not unique for these ORF's. Consequently, cross-hybridization is expected. The expression patterns of these ORF's are highly similar, especially for IMD1, IMD2 and IMD3. Twelve peptides could be retrieved which had 100% protein sequence similarity with one or more members of the IMD family. Amongst these twelve, there were several unique for one particular member of the IMD family (and also unique with respect to the rest of the genome).

Peptide nr	3	6	8	9	10	11
Unique for	IMD 1	IMD 2	IMD 3	IMD 3	IMD 3	IMD 3

Thus a unique peptide was found for YAR073W.

2. YGR069w

A unique peptide was found for YGR069w

3. YPR195C

A unique peptide was found for YPR195C

All alignments were obtained using BLAST [2]. Visualization of the alignment of all identified peptides is performed using ClustalX [3] (described in [4]) and is found on the following pages.

References:

[1] M.J.L. de Groot *et al.*, 2007, Quantitative proteomics and transcriptomics of anaerobic and aerobic yeast cultures reveals posttranscriptional regulation of key cellular processes, Microbiol – SGM

[2] NCBI BLAST, http://www.ncbi.nlm.nih.gov/blast/Blast.cgi

[3] ClustalX ftp://ftp-igbmc.u-strasbg.fr/pub/ClustalX/

[4] J.D. Thompson *et al.*, 1997, The ClustalX windows interface: flexible strategies for multiple sequence alignment aided by quality analysis tools. Nucleic Acids Research, 25:4876-4882.

List of the 54 dubious genes. The grey boxes indicate genes which are not found on YG 2.0, the successor of the Affymetrix YG S98 GeneChip.

'YAR069C'	'YER188W'	'YJL199C-MBB1'	'YML089C'
'YAR073W-IMD1'	'YFR020W'	'YKL031W'	'YML122C'
'YAR075W-IMD2'	'YGL042C'	'YKL066W'	'YMR052C-A'
'YBL048W'	'YGL188C'	'YKL076C-PSY1'	'YMR103C'
'YBL065W'	'YGR039W'	'YKL136W'	'YMR320W'
'YBL070C'	'YGR069W'	'YKL169C'	'YNL140C-RLR1'
'YBR056w-a'	'YGR073C'	'YKL177W'	'YOL153C'
'YBR116C-TKL2'	'YGR122C-A'	'YKR012C'	'YOL160W'
'YBR292C'	'YGR182C'	'YLL020C-KNS1'	'YOR013W'
'YBR300C'	'YGR190C'	'YLR112W'	'YOR318C'
'YCL012W-BUD3'	'YIL059C'	'YLR252W'	'YPR150W'
'YDR094W'	'YIL071w-a'	'YLR280C'	'YPR195C'
'YDR133C'	'YIL167W-SDL1'	'YLR294C'	
'YER067c-a'	'YJL119C'	'YLR311C'	

Peptide 1;

```
YAR075WP_042831 ---------------------------------------------------
YML065C_IMD4_YEAST EDDSLVVSEVMTKNPVTGIKGITLKEGNEILKQTKKGKLLIVDDNGNLVSM 225
YHR432W_IMD3_YEAST EDNSLLVQDVMTKNPVTGAQGITLSEGNEILKKIKKGKLLIVDDNGNLVSM 224
YHR216W_IMD2_YEAST EDNSLLVQDVMTKNPVTGAQGITLSEGNEILKKIKKGRLLVVDEKGNLVSM 224
YAR073W_IMD1_YEAST EDNSLLVQDVMTKNPVTGAQGITLSEGNEILKKIKKGRLLVVDEKGNLVSM 224
pep1 ------------KNPVTGAQGITLSEGNEILKK------------------ 21
ruler ...180.......190.......200.......210.......220.....
```

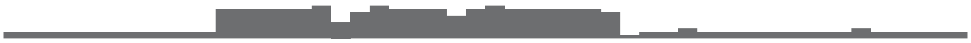

Peptide 2;

```
YAR075WP_042831 ---------------------------------------------------
YML065C_IMD4_YEAST EDDSLVVSEVMTKNPVTGIKGITLKEGNEILKQTKKGKLLIVDDNGNLVSM 225
YHR432W_IMD3_YEAST EDNSLLVQDVMTKNPVTGAQGITLSEGNEILKKIKKGKLLIVDDNGNLVSM 224
YHR216W_IMD2_YEAST EDNSLLVQDVMTKNPVTGAQGITLSEGNEILKKIKKGRLLVVDEKGNLVSM 224
YAR073W_IMD1_YEAST EDNSLLVQDVMTKNPVTGAQGITLSEGNEILKKIKKGRLLVVDEKGNLVSM 224
pep2 -------------------------------------RLLVVDEKG----- 9
ruler ...180.......190.......200.......210.......220.....
```

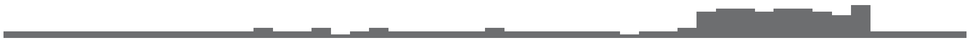

Peptide 3; unique for IMD1

```
                                   .    ..*.  *
YHR432W_IMD3_YEAST DLEIIAGNVATREQAANLIAAGADGLRIGMGSGSICITQEVMACGRPQGTA 110
YML065C_IMD4_YEAST DLEIIAGNVATREQAANLIAAGADGLRIGMGSGSICITQEVMACGRPQGTA 111
YHR216W_IMD2_YEAST GLEVIAGNVVTREQAANLIAAGADGLRIGMGTGSICITQEVMACGRPQGTA 110
YAR073W_IMD1_YEAST DLEIIAGNVVTKEQAANLIAAGADGLRIGMGTGSICITQKVMACGRPQGTA 350
YAR075WP_042831 RLKAYRGMGSIDAMQKTGTKGNASTSRYFSESDSVLVAQGVSGAVVDKGSI 95
pep3 -----------KEQAANLIAAGADGLRI----------------------- 17
ruler 0.......310.......320.......330.......340.......350
```

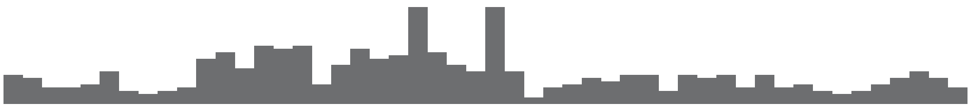

Peptide 4;

```
YAR075WP_042831 ---------------------------------------------------
YML065C_IMD4_YEAST VGEVKVMKRKFGFSGFPVTEDGKCPGKLVGLVTSRDIQFLEDDSLVVSEVM 185
YHR432W_IMD3_YEAST VGEAKSMKERFGFSGFPVTEDGKRNGKLMGIVTSRDIQFVEDNSLLVQDVM 184
YHR216W_IMD2_YEAST VGEAKSMKEKYGFAGFPVTTDGKRNAKLVGVITSRDIQFVEDNSLLVQDVM 184
YAR073W_IMD1_YEAST VGEAKSMKEKYGFAGFPVTADGKRNAKLVGAITSRDIQFVEDNSLLVQDVM 184
pep4 --------------------------KLVGVITSRD--------------- 10
ruler ...140.......150.......160.......170.......180.....
```

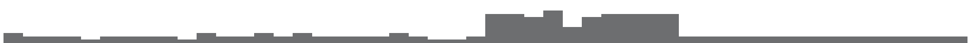

Peptide 5;

```
    YAR075WP_O42831 ---------------------------------------------------
YML065C_IMD4_YEAST IVDDNGNLVSMLSRADLMKNQNYPLASKSATTKQLLCGAAIGTIEADKERL   265
YHR432W_IMD3_YEAST IVDDNGNLVSMLSRTDLMKNQNYPLASKSATTKQLLCGAAIGTIDADKERL   264
YHR216W_IMD2_YEAST VVDEKGNLVSMLSRTDLMKNQNYPLASKSANTKQLLCGASIGTMDADKERL   264
YAR073W_IMD1_YEAST VVDEKGNLVSMLSRTDLMKNQKYPLASKSANTKQLLWGASIGTMDADKERL   264
              pep5 ------------------KNQNYPLASKS----------------------    11
             ruler ...220.......230.......240.......250.......260.....
```

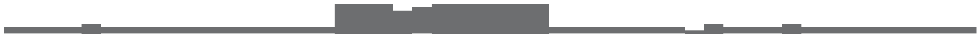

Peptide 6; unique for IMD2

```
YHR216W_IMD2_YEAST SIFELNMLKWVKESFPGLEVIAGNVVTREQAANLIAAGADGLRIGMGTGSI   334
              pep6 -----------KESFPGLEVIAGNVVTRE----------------------    18
YHR432W_IMD3_YEAST SIFQLNMIKWIKETFPDLEIIAGNVATREQAANLIAAGADGLRIGMGSGSI   334
YML065C_IMD4_YEAST SVFQLNMIKWIKETFPDLEIIAGNVATREQAANLIAAGADGLRIGMGSGSI   335
   YAR075WP_O42831 ------MVVF-----------------------------------------     4
YAR073W_IMD1_YEAST SIFQLNMIKWIKETFPDLEIIAGNVVT------------------------   310
             ruler ...290.......300.......310.......320.......330.....
```

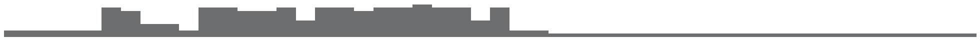

Peptide 7;

```
                                      * ***   **.
   YAR075WP_O42831 TESPGEYLYQDGKRLKAYRGMGSIDAMQKTGTKGNASTSRYFSESDSVLVA    82
YML065C_IMD4_YEAST TESPGEYFYKDGKRLKAYRGMGSIDAMQKTGNKGNASTSRYFSESDSVLVA   449
YHR432W_IMD3_YEAST TESPGEYFYQDGKRLKAYRGMGSIDAMQKTGTKGNASTSRYFSESDSVLVA   448
YHR216W_IMD2_YEAST TESPGEYFYQDGKRLKAYRGMGSIDAMQKTGTKGNASTSRYFSESDSVLVA   448
YAR073W_IMD1_YEAST KEQAANLIAAGADGLRIGMGTGSICITQKVMACGRPQGTAVYN---VCEFA   358
              pep7 ------------------RGMGSIDAMQKT---------------------    12
             ruler 0.......410.......420.......430.......440.......450
```

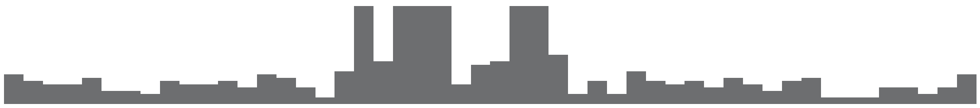

Peptide 8;

```
   YAR075WP_O42831 ---------------------------------------------------
YML065C_IMD4_YEAST MSAAPLDYKKALEHLKTYSSKDGLSVQELMDSTTRGGLTYNDFLVLPGLVN    51
YHR432W_IMD3_YEAST -MAAVRDYKTALEFAKSLPRLDGLSVQELMDSKTRGGLTYNDFLVLPGLVD    50
YHR216W_IMD2_YEAST -MAAIRDYKTALDFTKSLPRPDGLSVQELMDSKIRGGLTYNDFLILPGLVD    50
YAR073W_IMD1_YEAST -MAAIRDYKTALDLTKSLPRPDGLSVQELMDSKIRGGLAYNDFLILPGLVD    50
              pep8 -------------------RLDGLSVQELMDSKT-----------------    15
             ruler 1.......10........20........30........40........50.
```

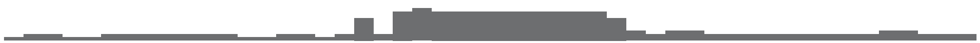

Peptide 9; unique for IMD3

```
  YAR075WP_042831 ---------------------------------------------------
YML065C_IMD4_YEAST FPVTEDGKCPGKLVGLVTSRDIQFLEDDSLVVSEVMTKNPVTGIKGITLKE   200
YHR432W_IMD3_YEAST FPVTEDGKRNGKLMGIVTSRDIQFVEDNSLLVQDVMTKNPVTGAQGITLSE   199
YHR216W_IMD2_YEAST FPVTTDGKRNAKLVGVITSRDIQFVEDNSLLVQDVMTKNPVTGAQGITLSE   199
YAR073W_IMD1_YEAST FPVTADGKRNAKLVGAITSRDIQFVEDNSLLVQDVMTKNPVTGAQGITLSE   199
             pep9 -----------KLMGIVTSRD------------------------------    10
            ruler 0.......160.......170.......180.......190.......200
```

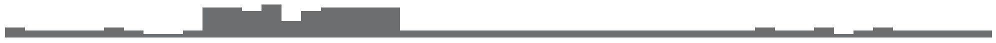

Peptide 10; unique for IMD3

```
  YAR075WP_042831 ---------------------------------------------------
YML065C_IMD4_YEAST EGNEILKQTKKGKLLIVDDNGNLVSMLSRADLMKNQNYPLASKSATTKQLL   250
YHR432W_IMD3_YEAST EGNEILKKIKKGKLLIVDDNGNLVSMLSRTDLMKNQNYPLASKSATTKQLL   249
YHR216W_IMD2_YEAST EGNEILKKIKKGRLLVVDEKGNLVSMLSRTDLMKNQNYPLASKSANTKQLL   249
YAR073W_IMD1_YEAST EGNEILKKIKKGRLLVVDEKGNLVSMLSRTDLMKNQKYPLASKSANTKQLL   249
            pep10 ------------KLLIVDDNGNLVSMLSRT---------------------    18
            ruler 0.......210.......220.......230.......240.......250
```

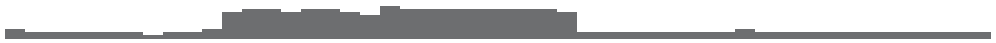

Peptide 11; unique for IMD3

```
  YAR075WP_042831 ---------------------------------------------------
YML065C_IMD4_YEAST QNYPLASKSATTKQLLCGAAIGTIEADKERLRLLVEAGLDVVILDSSQGNS   285
YHR432W_IMD3_YEAST QNYPLASKSATTKQLLCGAAIGTIDADKERLRLLVEAGLDVVILDSSQGNS   284
YHR216W_IMD2_YEAST QNYPLASKSANTKQLLCGASIGTMDADKERLRLLVKAGLDVVILDSSQGNS   284
YAR073W_IMD1_YEAST QKYPLASKSANTKQLLWGASIGTMDADKERLRLLVKAGLDVVILDSSQG--   282
            pep11 ------------KQLLCGAAIGTIDADKERL--------------------    19
            ruler ...240.......250.......260.......270.......280.....
```

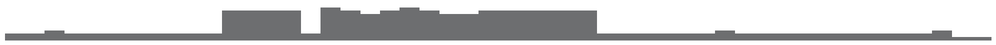

Peptide 12;

```
YHR432W_IMD3_YEAST IFQLNMIKWIKETFPDLEIIAGNVATREQAANLIAAGADGLRIGMGSGSIC   275
            pep12 ----------KETFPDLEIIAGNVATRE-----------------------    18
  YAR075WP_042831 ---------------------------------------------------     5
YML065C_IMD4_YEAST VFQLNMIKWIKETFPDLEIIAGNVATREQAANLIAAGADGLRIGMGSGSIC   276
YHR216W_IMD2_YEAST IFELNMLKWVKESFPGLEVIAGNVVTREQAANLIAAGADGLRIGMGTGSIC   335
YAR073W_IMD1_YEAST IFQLNMIKWIKETFPDLEIIAGNVVT-------------------------   310
            ruler ...290.......300.......310.......320.......330.....
```

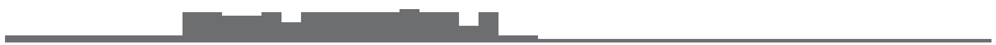

Peptide unique for YGR069W

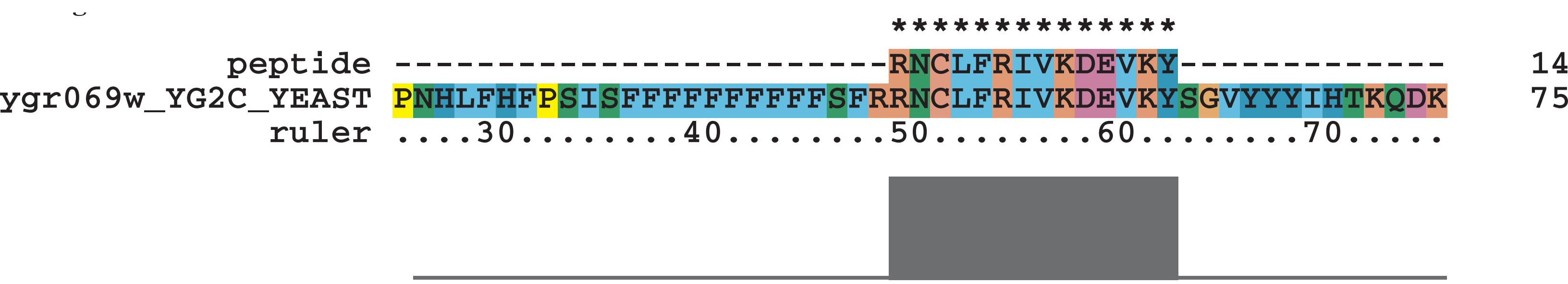

Peptide unique for YPR195C

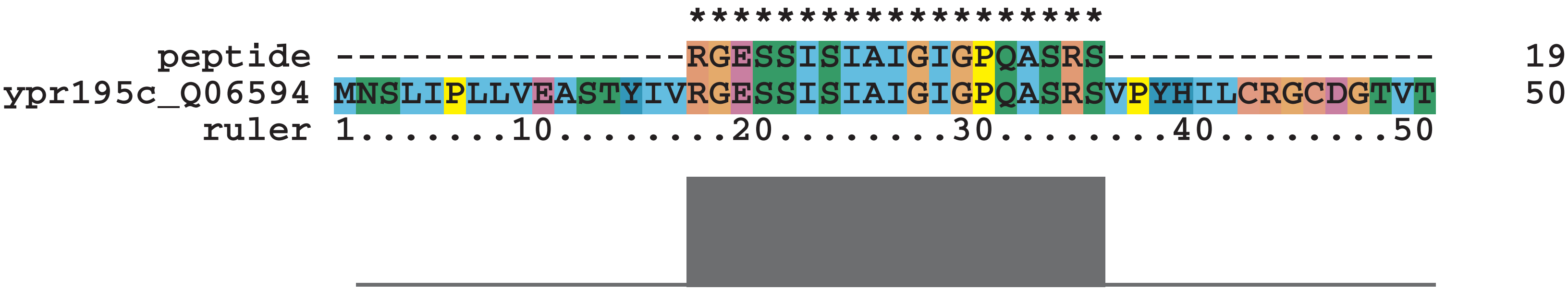

Supplement: Additional file 5 — Peptide quantification and BLAST results for dubious genes. This file provides information on the peptide quantification and BLAST results for three dubious genes. Also, it states which of the 54 dubious genes are not found on YG 2.0, the successor of the Affymetrix YG S98 GeneChip. [file 1471-2164-10-53-S5.pdf]
