# Supplementary material for: Combinatorial effects of environmental parameters on transcriptional regulation in Saccharomyces cerevisiae: A quantitative analysis of a compendium of chemostat-based transcriptome data
Source: BMC Genomics. 2009 Jan 27;10:53. doi: 10.1186/1471-2164-10-53 (PMC2640415; doi:10.1186/1471-2164-10-53)
Supplement: Additional file 7 — Interactive gene expression visualization tool. This file contains the download link of a self extractable executable that contains an application that allows one to visualize the expression patterns of genes across the compendium. The conditions within the compendium can be sorted on two of the ten cultivation parameters. It is possible to analyze the significant cultivation parameters selected by the regression model to reconstruct the gene expression patterns. The Matlab code for this visualization tool can be obtained via the corresponding author. [file 1471-2164-10-53-S7.pdf]

http://www-ict.ewi.tudelft.nl/~theo/CSSC_GeneVisualizer_pkg.exe (when Matlab R2006 is on your machine)
http://www-ict.ewi.tudelft.nl/~theo/CSSC_GeneVisualizer_mcr_pkg.exe (when Matlab R2006 is not on your machine).
