# Supplementary material for: Combinatorial effects of environmental parameters on transcriptional regulation in Saccharomyces cerevisiae: A quantitative analysis of a compendium of chemostat-based transcriptome data
Source: BMC Genomics. 2009 Jan 27;10:53. doi: 10.1186/1471-2164-10-53 (PMC2640415; doi:10.1186/1471-2164-10-53)
Supplement: Additional file 9 — Choice of the regression strategy. The choice for a step-wise regression approach is substantiated. [file 1471-2164-10-53-S9.pdf]

Additional File 9 - Choice of the regression strategy

The choice for the step-wise approach is based on the intended goal and the properties of the predictors. The goal was to explain the measured gene expression levels in terms of the (combinatorial) cultivation parameters, which form the predictors. It was expected that a gene responds to a relatively small subset of the predictors and it was considered important to identify this subset. There is a large number of correlated (i.e. partially redundant) predictors. Design matrix **D** contains 227 predictors, which is larger than the number of samples (170), making the problem under-determined with an infinite number of perfect solutions. Ordinary least squares (OLS) is not appropriate, because of the insufficient prediction accuracy in such situations [Tibshirani1996].

Approaches that use the singular value decomposition (SVD) or principal component analysis (PCA) to transform the predictor matrix into a lower dimensional orthogonal matrix such as principal component regression (PCR) and partial least squares regression (PLS) are not suitable, because the new predictors are linearly weighted sums of the 'actual' predictors, i.e. the cultivation parameters, and it is not straightforward to derive the influence and statistical significance of the 'actual' cultivation parameters from these models.

Shrinkage methods (e.g. RIDGE and LASSO) and selection approaches (e.g. stepwise) form another type of modeling approach in this situation [Tibshirani1996]. Although RIDGE regression shrinks coefficients, it does not set any coefficients to 0 and hence does not give an easily interpretable model. LASSO and selection approaches do select a subset of significant predictors. We have chosen for the step-wise approach, since Hastie et al. [Hastie2007] concludes that for problems with large numbers of correlated predictors the forward stepwise procedure might be preferable to the lasso approach *and* we could easily build in a leave-one-out cross-validation scheme to increase the robustness of the selected predictors. (This is to combat the drawback of variability in selection approaches, caused by its discrete nature, i.e. predictors are either retained or not [Tibshirani1996].)

[Tibshirani1996] *Regression selection and shrinkage via the lasso* R Tibshirani - J. Royal Statist. Soc. B, 1996

[Hastie2007] *Forward stagewise regression and the monotone lasso* T Hastie, J Taylor, R Tibshirani, G Walther - Electronic Journal of Statistics, 2007
