# Supplementary material for: Combinatorial effects of environmental parameters on transcriptional regulation in Saccharomyces cerevisiae: A quantitative analysis of a compendium of chemostat-based transcriptome data
Source: BMC Genomics. 2009 Jan 27;10:53. doi: 10.1186/1471-2164-10-53 (PMC2640415; doi:10.1186/1471-2164-10-53)
Supplement: Additional file 10 — Flowcharts of enrichment analysis. Page 1 – Flowchart of Methods – Enrichment analysis. All significant regression weights are stored in a large matrix. This matrix has 6005 rows, which represent all differentially expressed genes for which the regression analysis was performed, and 227 columns, which represent the (combinatorial) cultivation parameters, which were used as predictors in the regression model. An element of this matrix indicates the assigned regression weight of a cultivation parameter to explain the expression pattern of a gene. A zero indicates that the cultivation parameter was not selected as a significant predictor for a gene. For each column of this matrix, we group the genes that were upregulated (positive weights), downregulated (negative weights) or either up- or downregulated (non-zero weights) by the cultivation parameter related to that column. These groups are analyzed for significant overlap with gene groups derived from functional annotation databases and TF binding information using the hypergeometric test. The enrichment results are visually represented in Additional File 1. Page 2 – Flowchart of Methods – Functional categories specifically influenced by a combinatorial effect Gene groups are derived based on the large matrix with regression weights. In this case, for each combinatorial effect we create four gene groups: 1) We group the genes that respond to the combinatorial effect (non-zero weights). 2) We group the genes that respond to one of the single effects that constitute the combinatorial effect. 3) We group the genes that respond to the other single effect. 4) We group genes that respond to both single effects. Again, the hypergeometric test is employed to analyze these gene groups for significant overlap with gene groups derived from functional categories. Page 3 – Flowchart of Methods – Clustering of genes based on regression coefficients Firstly, genes that respond (non-zero weights) to a particular (combinatorial) culti [file 1471-2164-10-53-S10.pdf]

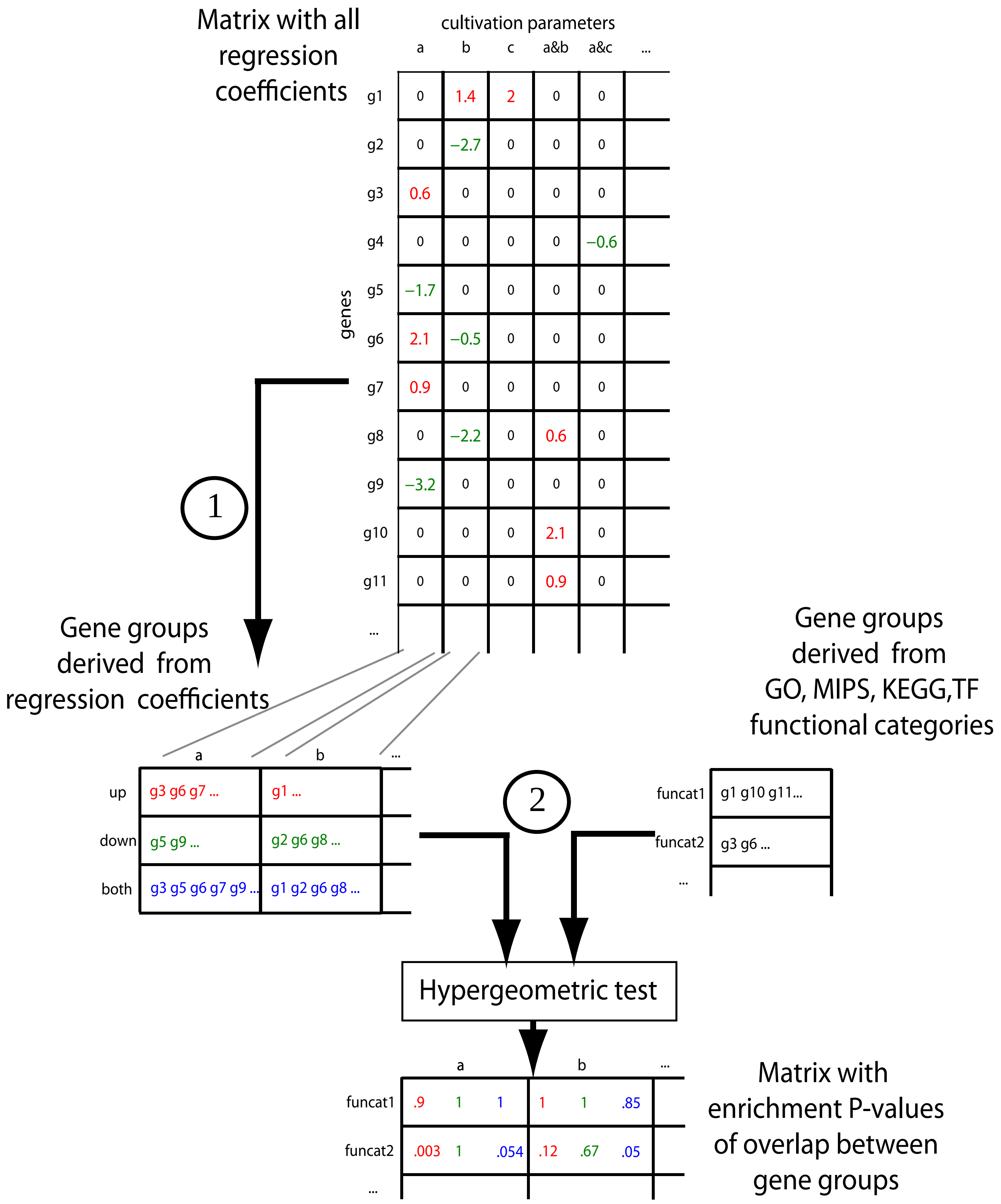

Flowchart of Methods - Enrichment analysis
All significant regression weights are stored in a large matrix. This matrix has 6005 rows, which represent all differentially expressed genes for which the regression analysis was performed, and 227 columns, which represent the (combinatorial) cultivation parameters, which were used as predictors in the regression model. An element of this matrix indicates the assigned regression weight of a cultivation parameter to explain the expression pattern of a gene. A zero indicates that the cultivation parameter was not selected as a significant predictor for a gene. Step 1: For each column of this matrix, we group the genes that were upregulated (positive weights), downregulated (negative weights) or either up- or downregulated (non-zero weights) by the cultivation parameter related to that column. Step 2: These groups are analyzed for significant overlap with gene groups derived from functional annotation databases and TF binding information using the hypergeometric test. The enrichment results are visually represented in Additional File 2.

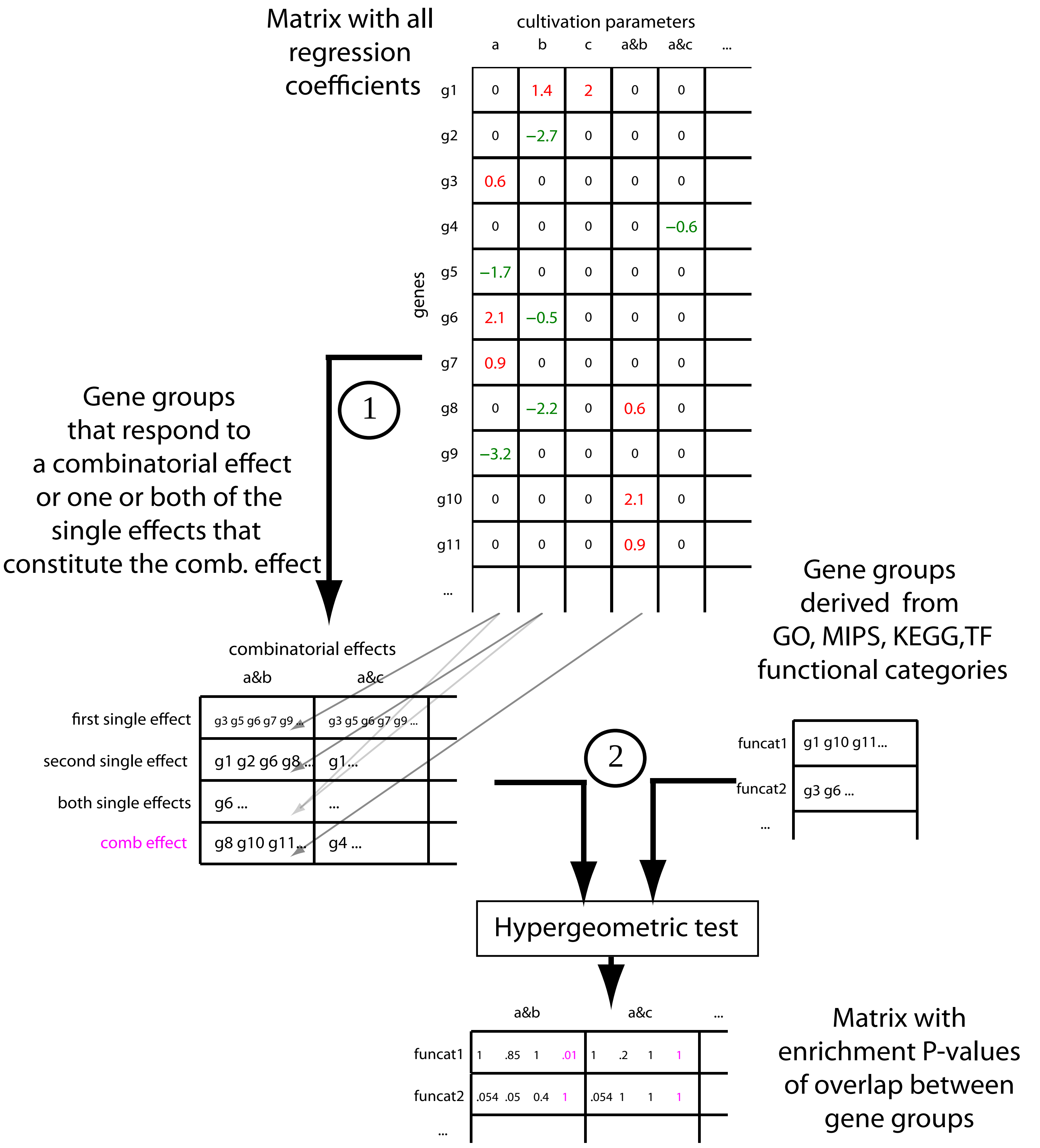

Flowchart of Methods - Functional categories specifically influenced by a combinatorial effect
Step 1: Gene groups are derived based on the large matrix with regression weights. In this case, for each combinatorial effect we create four gene groups: 1) We group the genes that respond to the combinatorial effect (non-zero weights). 2) We group the genes that respond to one of the single effects that constitute the combinatorial effect. 3) We group the genes that respond to the other single effect. 4) We group genes that respond to both single effects. Step 2: Again, the hypergeometric test is employed to analyze these gene groups for significant overlap with gene groups derived from functional categories.

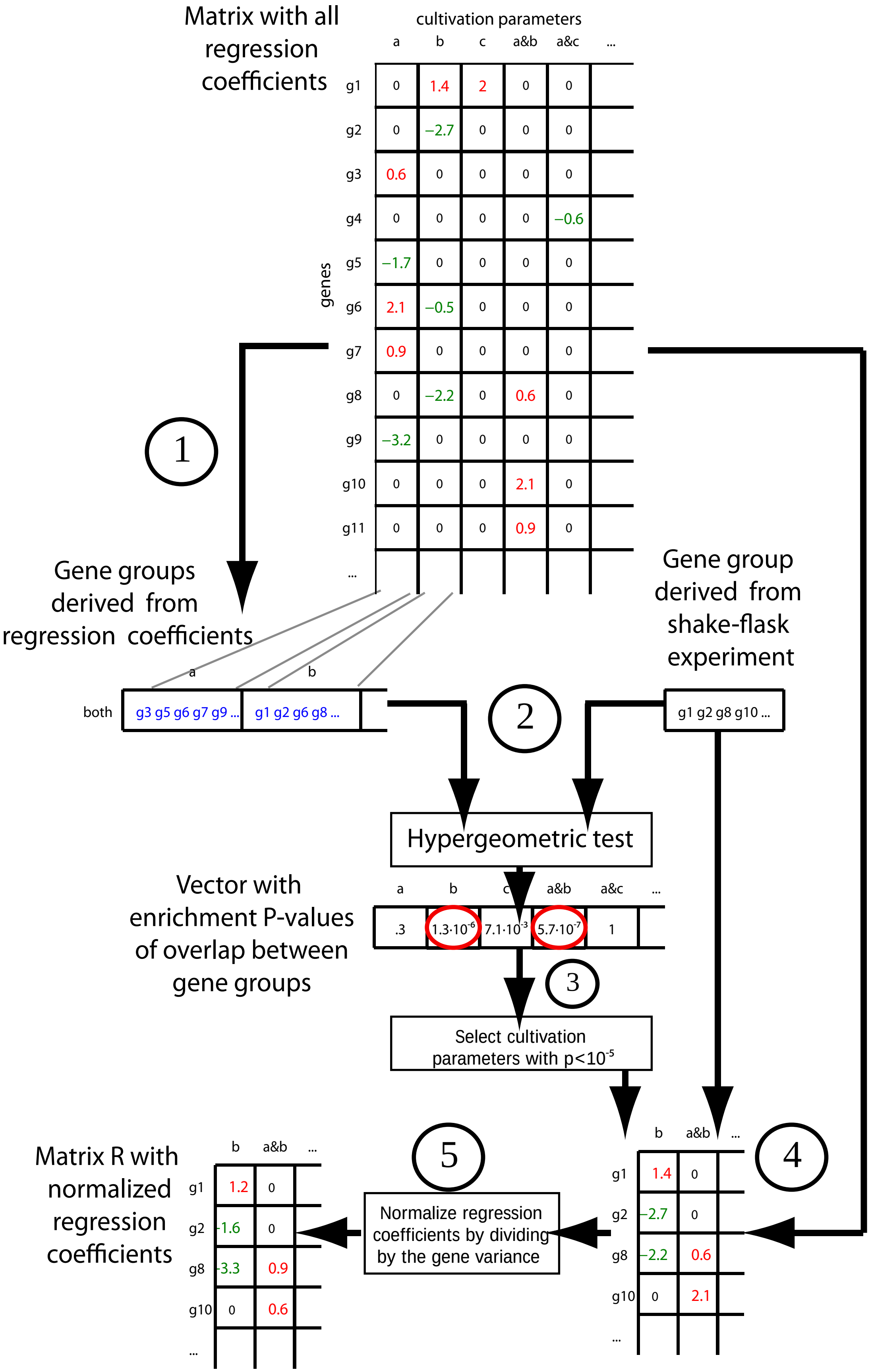

Flowchart of Methods - Clustering of genes based on regression coefficients
Step 1: Firstly, genes that respond (non-zero weights) to a particular (combinatorial) cultivation parameter are grouped. (These groups are identical to the blue groups generated in the enrichment analysis flowchart on Page 1 of this document.)
Step 2: These groups are analyzed for significant overlap with the gene group derived from the shake-flask experiment.
Step 3: The significant cultivation parameters are selected. (These are the cultivation parameters to which significantly many of the genes in the shake-flask gene group respond.) Step 4: A new matrix is derived. This matrix contains the regression weights of the genes of the shake-flask gene group for the signficant cultivation parameters. Bascially, this matrix is a submatrix of the original regression weight matrix. Step 5: By normalizing these regression weights, we arrive at matrix **R**, which is used in the subsequent cluster analysis.
